# Impact of motor guided imagery therapy on stroke patients with pain symptoms: a three-level meta-analysis and systematic review of randomized controlled trials

**DOI:** 10.3389/fneur.2026.1920523

**Published:** 2026-10-06

**Authors:** Xinmo Li, Hong Ni, Guangze Cui, Chen Wei, Yongguo Zhu

**Affiliations:** 1 Institute of Physical Education and Training, Capital University of Physical Education and Sports, Beijing, China; 2 School of Recreation and Community Sport, Capital University of Physical Education and Sports, Beijing, China; 3 The High School Affiliated to Beijing Jiaotong University, Beijing, China, 3Shenyang Normal University, Beijing, China; 4 College of Sports Science, Shenyang Normal University, Shenyang, China; 5 School of Ice‑Snow Sports, Capital University of Physical Education and Sports, Beijing, China

**Keywords:** motor guided imagery, neurorehabilitation, post-stroke pain, stroke, three-level meta-analysis

## Abstract

**Background:**

Post-stroke pain is a common and intractable complication that hinders neurorehabilitation progress. Motor Guided Imagery (MGI), an emerging rehabilitation technique based on brain-computer interaction principles, has shown potential in relieving post-stroke pain, yet high-quality evidence on its multi-dimensional efficacy remains insufficient. This study aimed to systematically evaluate the effects of MGI on pain, sensorimotor function, sleep quality, life status, and psychological mood in stroke patients using a three-level meta-analysis.

**Objective:**

This study aimed to quantitatively assess the therapeutic effects of Motor Guided Imagery for stroke-related pain and explore potential intervention moderators using a three-level meta-analysis framework.

**Methods:**

Eight Chinese and English databases were searched from inception to June 29, 2025, to collect randomized controlled trials (RCTs) of MGI for stroke patients with pain. A three-level random-effects model was adopted to pool Hedges’ *g* effect sizes, accompanied by heterogeneity testing, moderator analysis, influence analysis, and publication bias assessment. Influence-based outlier detection was implemented; analyses retaining all effect-size observations were treated as the primary main analysis, while outlier-removed analyses were reported only as supplementary sensitivity checks.

**Results:**

Motor-guided imagery training significantly relieved pain (*g* = −0.524, 95% CI -0.779 to −0.269, *p* = 0.0006; negative Hedges’ g indicates clinical improvement for this outcome) and improved sleep quality (*g* = −1.658, 95% CI -2.115 to −1.201, *p* < 0.001; negative Hedges’ g indicates clinical improvement for this outcome) in stroke patients. Meanwhile, it significantly enhanced motor function (*g* = 1.395, 95% CI 0.963 to 1.826, *p* < 0.001), life status (*g* = 1.757, 95% CI 0.966 to 2.549, *p* = 0.0012), and psychological-emotional status (*g* = 1.729, 95% CI 1.045 to 2.413, *p* = 0.0002); positive Hedges’ g indicates clinical improvement for the latter three outcomes. Moderator analysis indicated that acute-phase intervention (≤4 weeks) and high-frequency regimens (>5 sessions/week) tended to produce better analgesic effects, while age, gender, single-session duration, and pain location had no significant moderating effects.

**Conclusion:**

MGI may produce beneficial rehabilitation effects that can alleviate post-stroke pain and synergistically improve motor function, sleep, daily-living ability, and psychological state of stroke patients. Exploratory subgroup trends indicated potentially larger effects for early implementation within the acute phase and high-frequency short-duration regimens. However, these observations should be interpreted as hypothesis-generating clues given non-significant overall moderator tests. Further high-quality research is required to confirm these findings.

**Registration:**

The systematic review protocol was retrospectively registered with PROSPERO under registration ID CRD420261278812, after literature search and full-text screening had been completed.

**Systematic review registration:**

https://www.crd.york.ac.uk/PROSPERO/view/CRD420261278812; identifier: CRD420261278812

## 1 Introduction

Post-stroke pain is a highly prevalent complication among stroke survivors, occurring in 19–47% of patients and impairing rehabilitation prognosis and daily-life quality across physical sensation, limb motor performance, sleep, and psychological mood domains (1). Clinically, this condition is frequently overshadowed by overt stroke-related deficits such as motor or aphasic impairments, leading to under-recognition and inadequate intervention (2). Persistent post-stroke pain is not merely an isolated physical complaint; it can further exacerbate functional impairment and trigger secondary sequelae including insomnia, anxiety, and depression. This pain often manifests as hemibody paresthesia, burning, or stabbing sensations and is frequently accompanied by psychological issues such as anxiety and depression. This creates a vicious cycle of “pain → dysfunction → psychological disorder,” constituting a key bottleneck hindering rehabilitation progress. Furthermore, Shoulder-Hand Syndrome (SHS), a highly representative post-stroke complication, has an incidence ranging from 12.5 to 70.0%. Approximately 90% of SHS patients experience severe shoulder pain, characterized by persistent burning and tenderness in the shoulder, which may radiate to the forearm and hand, severely limiting upper limb mobility (3). This pain often coexists with limb swelling and restricted joint movement, forming another vicious cycle of “pain → immobilization → functional decline.” This leads to loss of daily living abilities, stalls rehabilitation, and is a central obstacle to upper limb functional recovery after stroke (4).

Motor Guided Imagery (MGI) is an emerging rehabilitation intervention technique. Its core operational feature is that participants mentally simulate limb movements guided by audio-scripts or video prompts, without performing actual physical limb movements. Mirror therapy and action-observation-only therapy are excluded from the definition of MGI in the present study. It operates through a dual mechanism of “visual guidance + motor imagery simulation” to form virtual motor representations in the brain’s motor cortex. This process can both activate plasticity in damaged neural pathways and exert analgesic effects by modulating central pain conduction pathways (5, 6). Compared with conventional rehabilitation therapies, this technique requires no specialized equipment and is not limited by patients’ physical motor capacity, making it particularly suitable for stroke patients who cannot perform active training due to pain or severe motor-function impairment (7). Clinical studies have confirmed that MGI training can reduce the Visual Analogue Scale (VAS) score by 2.1–3.5 points, improve the Pittsburgh Sleep Quality Index (PSQI) score by 30–40%, and significantly increase serum serotonin (5-HT) levels in stroke patients (8), thereby inhibiting central sensitization of pain signals.

Despite preliminary progress in the application of MGI in stroke rehabilitation, evidence regarding its analgesic efficacy remains inconsistent and constrained by methodological limitations. Firstly, existing systematic reviews have predominantly focused on motor function outcomes. High-quality meta-analyses specifically targeting post-stroke pain are relatively scarce, and their conclusions have not been consistent. For instance, a meta-analysis of 7 randomized controlled trials (RCTs) demonstrated that motor imagery interventions significantly reduced pain intensity in post-stroke patients (standardized mean difference, SMD = −0.62, *p* < 0.001) (9). However, a systematic review noted that due to substantial heterogeneity (*I*^2^ = 68%) among included studies regarding pain types, assessment tools, and intervention protocols, the efficacy of motor imagery for post-stroke pain still requires further validation with higher-quality research (10, 11). Secondly, intervention protocols lack standardization. Parameters such as training frequency (1–3 times/day), session duration (15–45 min/session), and guidance modality (audio/video/virtual reality) vary substantially across existing studies, making it difficult to establish best-practice guidelines (12, 13). Thirdly, research into the underlying mechanisms remains superficial. Most studies have only observed changes in clinical indicators, lacking exploration into alterations in functional connectivity within pain-related brain regions, such as the default mode network and insular cortex (14). Fourthly, the selection of outcome measures lacks systematicity and specificity. Existing studies often focus disjointedly on improvement in a single dimension and fail to establish a corresponding evaluation system targeting the core pathophysiological chain in post-stroke pain patients: “pain → dysfunction → psychological disorder → sleep impairment.” Clinical research has confirmed that post-stroke pain directly restricts limb movement, leading to functional impairment, while long-term functional limitation can result in psychological issues like anxiety and depression (15). Concurrently, pain and sleep disorders exacerbate each other; sleep deprivation further lowers pain thresholds, and psychological stress amplifies pain perception, forming a vicious cycle (16). This pathological chain aligns closely with the core framework of the International Classification of Functioning, Disability, and Health (ICF): “Body Function → Activity → Participation” (16). The ICF framework was adopted to guide our outcome-selection logic, as it provides a standardized, biopsychosocial-oriented classification system covering impairment of body function, limitations in activity, and restrictions in social participation after stroke. Each of our five predefined outcome domains maps onto distinct ICF dimensions: pain intensity, sleep quality and emotional state correspond to Body-function dimension; pain-related dysfunction reflects activity-level limitation; and the composite “life status” domain captures both activity and participation-related constructs. Therefore, this study specifically selected five core outcome indicators: Pain intensity (VAS/NRS scores) to directly reflect the core analgesic effect; Pain-related dysfunction (MPI score) to assess improvement in limb activity; Sleep quality (PSQI score) to disrupt the “pain-insomnia” interplay; Life status (Barthel Index/MBI/ADL scores) to measure the recovery of daily living activities; Emotional state (HADS score) to address the adverse psychological impact. These indicators form a complete evaluation chain of “symptom control → functional recovery → mind-body synergy,” precisely corresponding to patients’ pathophysiological characteristics while comprehensively covering the core objectives of rehabilitation intervention, thereby addressing the gap of unsystematic outcome assessment in existing research.

This study aims to integrate pain-subtype stratification analysis with a three-level meta-analysis. For the nested data structure where multiple effect sizes are reported within a single included study, subgroup analyses were performed according to pain type (shoulder pain versus mixed pain). By decomposing sampling variance, the sources of heterogeneity were precisely identified, resolving the problem of overestimated heterogeneity in conventional two-level analyses caused by ignoring the non-independence of effect sizes. This study further explored the moderating effects of parameters including intervention frequency, single-session duration and treatment course on therapeutic efficacy. It provides high-level evidence for the standardized application of MGI in post-stroke pain management and contributes to improved rehabilitation prognoses for patients.

## 2 Materials and methods

This study adhered to the Preferred Reporting Items for Systematic Reviews and Meta-Analyses (PRISMA) guidelines (17) for the selection and application of research methods and has obtained retrospective registration on the International Prospective Register of Systematic Reviews (PROSPERO) (CRD420261278812). This systematic review obtained retrospective PROSPERO registration after completing literature search and full-text screening. Analyses are therefore not prespecified but consistent with the retrospectively registered record. Decisions made before registration covered the PICO framework, search strategy and outcome grouping. Post-registration methodological choices included the three-level meta-analysis for nested non-independent effect sizes, moderator-analysis implementation rules, and influence-statistic-based outlier detection and handling. All post-registration analytical choices are fully reported in the manuscript.

### 2.1 Study framework

The PICO framework for this systematic review is presented in Table 1.

**Table 1:** PICO framework for the impact of motor guided imagery training on stroke patients with pain.

PICO element	Description	ICF corresponding dimension and code
Population	Diagnosed with stroke (ICD-10 or I60-I69) Presence of post-stroke pain (VAS > 0) • Exclusion of severe comorbid somatic diseases	Body function: Pain function (b280) Emotional function (b152) Movement function (b730)
Intervention	Type: MGI training (audio/video guidance + motor imagery simulation) Intensity: Low to moderate (RPE 3–5) Dosage: 15–45 min/session, 3–7 sessions/week, for 2–8 weeks Provider: Rehabilitation therapist/specialized trainer	Activity and participation: Exercise training (d455) Rehabilitation training (d460) Leisure activities (d920)
Comparison	Conventional rehabilitation (e.g., passive joint mobilization, physical agent therapy) • Control intervention without MGI	Environmental factors: Health services (e580) Rehabilitation services (e585)
Outcome	Primary: Pain intensity (VAS/NRS score → b280) Pain-related dysfunction (MPI score → d450) Secondary: Sleep quality (PSQI score → b400) Life status (Barthel Index / MBI / ADL score → d640-d670) Emotional state (HADS score → b152)	ICF codes for outcomes: Pain function (b280) Emotional function (b152) Sleep function (b400) Daily life activities (d640-d670) Movement-related activities (d450)

### 2.2 Search strategy

We performed computerized searches in databases including PubMed, Cochrane Library, Web of Science, Embase, China National Knowledge Infrastructure (CNKI), Wanfang Data, China Biology Medicine (CBM) and VIP Database for literature on MGI applied to stroke patients with pain. The search period extended from database inception to June 29, 2025. Grey literature was not searched. Only peer-reviewed journal articles published in English or Chinese were included. The search methodology combined subject headings and free-text terms, and the reference lists of relevant articles were also screened. The full search strategy for the Web of Science database is presented below:

#1 TS = (Stroke OR “Cerebrovascular accident” OR “Ischemic stroke” OR “Hemorrhagic stroke” OR Post-stroke OR “Cerebral infarction” OR Hemiplegia OR Hemiparesis).

#2 TS = (“Motor imagery” OR “Guided motor imagery” OR “Kinesthetic imagery” OR “Mental practice” OR “Motor imagery training” OR “Motor Imagery Therapy”).

#3 TS = (Pain OR “Chronic pain” OR “Neuropathic pain” OR “Central Post-Stroke Pain” OR CPSP OR “Shoulder Pain” OR “Spastic Pain”).

#4 #1 AND #2 AND #3 NOT Preprint Citation Index.

Search strategies for all other databases are available in the Supplementary Table 1.

### 2.3 Inclusion and exclusion criteria

Inclusion criteria: (1) Published randomized controlled trials (RCTs) investigating MGI for treating pain symptoms in stroke patients (18); (2) Patients meeting stroke diagnostic criteria (ICD-10 I60-I69) (19) and reporting pain (VAS > 0) (20); disease course unrestricted; comparable baseline characteristics; (3) Two forms of Motor Guided Imagery (MGI) were eligible in this review: ① Stand-alone MGI: kinesthetic motor imagery guided by audio or written script without additional adjuvant treatments; ② Combined-form MGI: MGI superimposed upon background treatments including conventional rehabilitation, electrotherapy, or acupuncture.

Only one eligible comparison was selected per multi-arm study to avoid duplicate sampling from the same source. This approach increased the number of effect-size observations and improved statistical power.

(4) Studies using at least one of the following validated assessment tools: Visual Analogue Scale (VAS) (21) or Numeric Rating Scale (NRS) (22) for pain intensity; Fugl-Meyer Assessment (FMA) (23) for upper limb motor function; goniometry for shoulder range of motion (ROM) (24); Barthel Index (BI) or Modified Barthel Index (MBI) (25) for activities of daily living (ADL); Pittsburgh Sleep Quality Index (PSQI) (26) for sleep quality; and Hospital Anxiety and Depression Scale (HADS) (27), Hamilton Depression Rating Scale (HAMD) (28), or Hamilton Anxiety Rating Scale (HAMA) (29) for psychological state.

Exclusion criteria: (1) Non-RCTs (e.g., case reports, clinical observations); (2) Duplicate publications; (3) Incomplete data or inability to obtain complete raw data; (4) Studies dominated by mirror-therapy or pure action-observation therapy without kinesthetic motor rehearsal.

### 2.4 Literature screening and data extraction

Retrieved records were imported into EndNote 21 software for duplicate removal. Potentially eligible studies were screened based on titles/abstracts and full texts according to the inclusion criteria. Two researchers independently extracted relevant data from included studies using a pre-designed form, followed by cross-checking. Any discrepancies were resolved through discussion or consultation with a third researcher. Inter-rater reliability was maintained (Kappa coefficient ≥0.8).

### 2.5 Risk of bias assessment

This study adopted the RoB 2.0 risk-of-bias tool recommended by the Cochrane Collaboration (30) to assess the risk of bias of included studies. The assessment covers five domains: bias arising from the randomization process, bias due to deviations from intended interventions, bias due to missing outcome data, bias in measurement of the outcome, and bias in selection of the reported result. The risk of bias is categorized into three levels: low risk, some concerns, and high risk.

Additionally, the Grading of Recommendations Assessment, Development, and Evaluation (GRADE) framework (31) was used to evaluate the certainty of evidence for each outcome, classifying it as high, moderate, low, or very low. The initial certainty for RCTs is high and low for observational studies. Factors that may downgrade the certainty include risk of bias (e.g., inadequate allocation concealment), inconsistency (I^2^ > 50%), indirectness (e.g., population mismatch), imprecision (wide confidence intervals), and publication bias (each factor can lower certainty by 1 or 2 levels). Factors that may upgrade the certainty include large effect size (RR > 2 or < 0.5), dose–response gradient, and plausible confounding (each can raise certainty by 1 level) (32). The final certainty levels indicate: high (confidence that the true effect is close to the estimated effect), moderate (true effect is likely close, but may be substantially different), low (true effect may be substantially different), and very low (true effect is likely substantially different). This assessment provides a reliable evidence base for clinical decision-making. Two researchers independently conducted the GRADE assessment, with disagreements resolved by consensus involving a third researcher.

### 2.6 Statistical analysis

#### 2.6.1 Calculation of effect sizes

For the current study, means, standard deviations, and sample sizes from each included trial were extracted to calculate Hedges’ g (standardized mean difference, SMD) for each independent comparison. Individual-level Hedges’ g values and their corresponding sampling variances were calculated via the escalc(measure = “SMD”) function from the metafor R-package (version 4.4.1) (33, 34). The computational formula for Hedges’ g is given below (35):

g=[(Mt−Mc)/Sp]×J

J=1–3/[4(Nt+Nc)−9]

Where Mt. and Mc are post-intervention mean values for the intervention and control groups, respectively. Sp denotes the pooled standard deviation:

$$\mathrm{Sp}=\text{SQRT}[((\mathrm{Nt}-1){\mathrm{St}}^{2}+(\mathrm{Nc}-1){\mathrm{Sc}}^{2})/(\mathrm{Nt}+\mathrm{Nc}-2)]$$

Nt and Nc are sample sizes of intervention and control groups; St^2^ and Sc^2^ are the variances of two groups. J is the small-sample bias-correction factor.

All effect sizes were calculated based on post-intervention observed values. Multiple comparison groups within a single publication were treated as separate effect-size entries. Scales were categorized according to their scoring properties: lower-is-better scales including pain VAS/NRS, PSQI sleep scale, and HAMA-, HAMD-, and HADS-series scales; and higher-is-better function/activity-of-daily-living scales (FMA, MBI/ADL, and the psychological-function subscale of GQOLI-74). Following recommendations from the Cochrane Handbook for Systematic Reviews of Interventions (36), full-scale uniform sign correction was not performed for all lower-is-better scales. Raw effect-size outputs for pain VAS/NRS and the PSQI sleep scale were retained, where negative Hedges’ g indicates clinical improvement. To harmonize the interpretation of the composite psychological-emotional outcome, manual sign inversion (multiplied by −1) was applied to effect sizes of HAMA, HAMD, HADS-A, HADS-D, and total HADS after computation via escalc() and prior to input into the three-level model. No sign inversion was conducted for the psychological-function subscale of GQOLI-74, a positive-scoring quality-of-life instrument. After pre-processing, positive Hedges’ g indicates clinical improvement for psychological-emotional, motor-function, and life-status outcomes.

After computing individual effect-size estimates (yi) and their sampling variances (vi) for each comparison, these pre-calculated values were submitted to a three-level random-effects meta-analysis model to synthesize pooled effects for non-independent effect sizes. Magnitude thresholds for Hedges’ g were interpreted as small (|*g*| < 0.2), medium (0.2 ≤ |*g*| < 0.8), and large (|*g*| ≥ 0.8) (35).

#### 2.6.2 Three-level meta-analysis specification, sensitivity-analysis and outlier handling

For each separate outcome domain, a three-level random-effects meta-analysis (37) was fitted via the `rma.mv()` function in metafor (34), to account for statistically non-independent effect sizes originating from the same primary study.

The covariance structure was specified with nested random intercepts: `random = ~ 1 | study_id / effect_id`.

- Level 1: Known sampling variances of each individual effect size, supplied via the variance–covariance matrix argument `V`.
- Level 2: Within-study random-intercept variance, capturing covariance among multiple effect sizes nested within one study. “This three-level specification implicitly models the correlation among dependent effect sizes from the same study; no fixed manual within-study correlation coefficient was manually set by the user”. The magnitude of within-study correlation is determined empirically from the observed data via the estimated Level-2 variance component.
- Level 3: Between-study random-intercept variance representing true heterogeneity across independent RCTs.

Restricted maximum-likelihood (REML) was used for variance-component estimation. One-sided log-likelihood ratio tests were applied to evaluate whether the variance components at Level 2 and Level 3 were statistically significant.

Sensitivity and influence diagnostics were conducted for every outcome: leave-one-out influence analysis, studentized residuals (rstudent), Cook’s distance, and DFFITS statistics were calculated to detect influential observations and potential outliers.

Two sets of meta-analytic results are fully reported for outcomes with detected outliers:

1. Primary main analysis: analysis retaining all originally extracted effect-size observations (including potential outliers);
2. Sensitivity analysis: analysis after removal of outlier effect-size points flagged by post-hoc influence diagnostics, performed only to assess result robustness.

We report both sets of pooled effect-size estimates, 95% confidence intervals and heterogeneity statistics in the results section and supplementary material to allow direct comparison.

#### 2.6.3 Moderator analyses

To explore potential moderators of MGI efficacy for post-stroke pain and related functional-psychological outcomes, moderator analyses were performed under the three-level mixed-effects framework. Moderators included intervention type, intervention frequency, single-session duration, and total treatment course. Pooled effect sizes with 95% CIs were estimated within each subgroup. Within-subgroup significance was evaluated against zero effect. Between-group differences were tested, with the corresponding *P*-between value reported. A *P*-between < 0.05 was suggestive of a potential significant moderating effect. Notably, a statistically significant finding within one subgroup accompanied by a non-significant result in another subgroup cannot constitute conclusive evidence for genuine subgroup divergence.

#### 2.6.4 Publication-bias assessment

Funnel plots were generated for visual inspection of small-study effects. Egger’s regression test, in which the standard error was specified as the predictor, was implemented within the mixed-effects meta-analytic framework to detect funnel-plot asymmetry. The trim-and-fill method was additionally applied as an exploratory tool. An Egger’s test *p* < 0.05 indicated statistical evidence of funnel-plot asymmetry; *p* ≥ 0.05 denoted no detectable asymmetry. Importantly, these tests possess limited statistical power when the number of included studies is small; a non-significant result cannot rule out underlying publication bias.

All statistical analyses and visualizations were performed using R software (version 4.4.1), with the metafor package as the major analytical tool. The overall statistical significance threshold was set at *p* < 0.05.

## 3 Results

### 3.1 Characteristics of included studies

Adhering to the PRISMA 2020 statement (17), a systematic search was conducted in PubMed, Cochrane Library, Embase, Web of Science, CNKI, CBM, Wanfang, and VIP databases from inception to 29 June 2025, and the full-text screening process was finished on 31 October 2025. The initial search yielded 917 records. After duplicate removal, 848 records underwent title-abstract screening and 719 were excluded. A total of 131 articles entered full-text assessment. Ten articles could not be obtained despite retrieval attempts including university library resources, backward reference snowballing, and e-mail contact with corresponding authors. These records were excluded for unavailable full texts. A total of 13 publications reporting data from 11 unique independent RCTs involving 1,032 stroke patients were ultimately included. The study-selection flowchart (Figure 1) counts publications rather than unique trials; three of these publications represent derived reports originating from one single parent RCT with overlapping participants.

**Figure 1:**
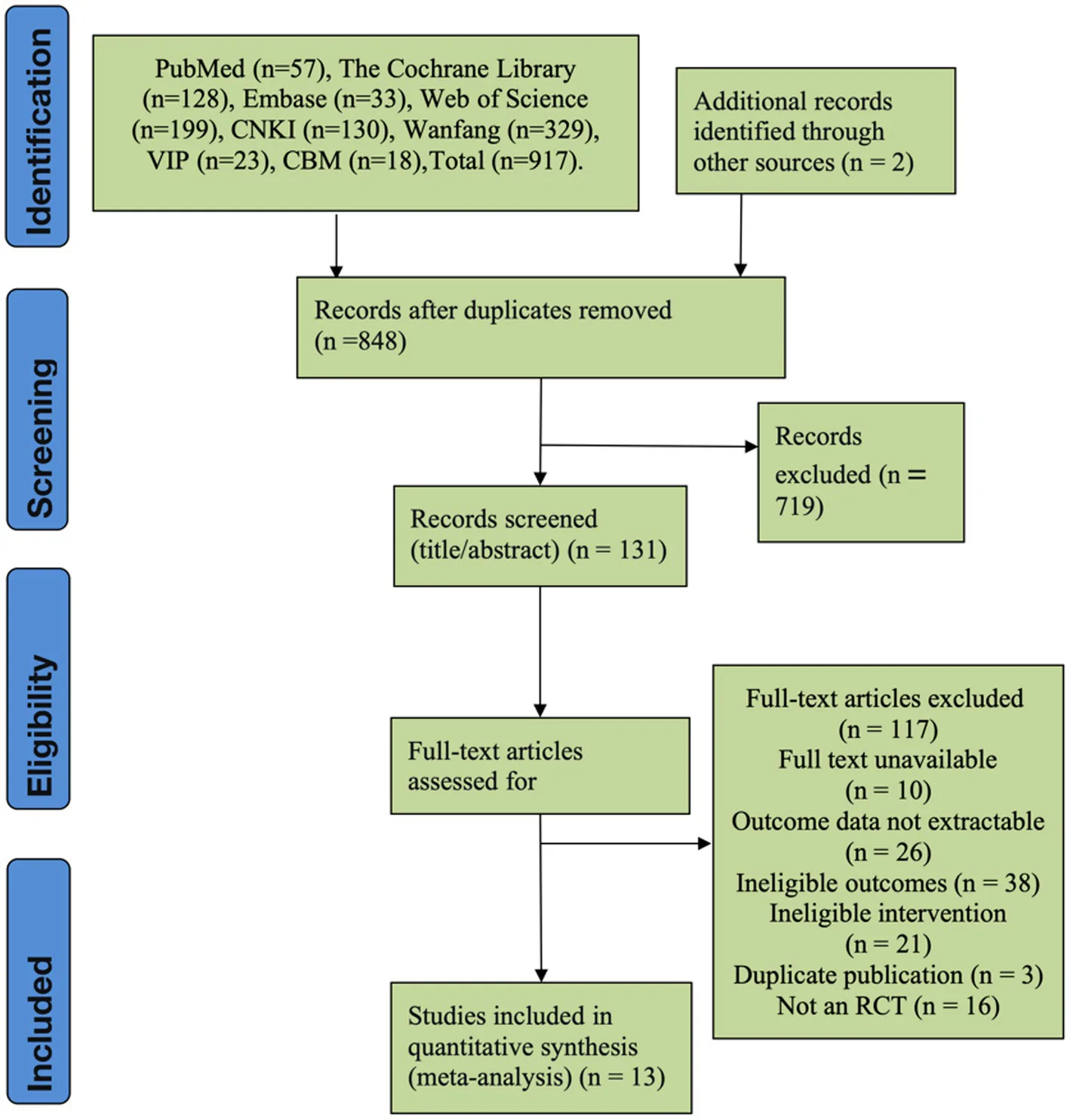
Flowchart of study selection.

The basic characteristics of the included studies are summarized in Table 2. The 13 studies included 1032participants (511 in experimental groups and 511 in control groups). All participants were stroke patients, and the publication years of the studies ranged from 2012 to 2024. The experimental groups received either MGI alone or MGI combined with other therapies (5 studies used combined interventions, 8 used MGI alone). The control groups received conventional rehabilitation therapy. Detailed intervention parameters, including modality, intensity, duration, frequency, and total time, were reported. Outcome measures covered pain, function, sleep quality, life status, and psychological indicators.

**Table 2:** Characteristics of included studies.

Study	Sample size	Gender (M/F)		Age (years)	Disease duration (days)			Pain type	Intervention	Intervention duration, frequency, course	Assessment tools*
	T/C		T/C		T/C		T/C		T/C		
Zhang. SS 2024 (52)	75/75	40/35	39/36	62.14 ± 3.51	61.85 ± 3.37	13.14 ± 2.25	13.05 ± 2.21	SHS	A + C/A	• Motor imagery therapy: 2 sessions/day. • Staged resistance training: 2–3 sets/day, 5 days/week. • Total course: 6 weeks. • Per session: 2–3 min relaxation + 5–7 min guided motor imagery.	① ④ ⑨
Xiao. YH 2024 (53)	20/20	13/7	14/6	61.35 ± 4.97	61.85 ± 4.82	54.95 ± 13.80	53.65 ± 14.51	SHS	A + C/A	• CT: 1 session/day, 5 days/week. • Electroacupuncture (EA): 1 session/day, 5 days/week. • Motor imagery therapy: 1 session/day, 5 days/week. • Total course: 2 weeks. Per session (MGI): ~30 min (1–2 min relaxation + 6–8 min upper limb imagery + 1–2 min attention focus, repeated 3 cycles).	② ④ ⑤
Jin. D 2022 (54)	46/46	29/19	27/19	59.16 ± 5.30	59.10 ± 5.25	29.21 ± 6.48	29.18 ± 6.52	SHS	A + C/A	• Rehabilitation therapy: 1 session/day. • TENS: 1 session/day. • Motor imagery therapy: 1 session/day. • Total course: 4 weeks. • Per session (MGI): ~10 min (3 min relaxation + 5 min imagery + 2 min attention focus).	③ ⑥
Gao. Z 2021 (55)	41/41	34/7	32/9	56.00 ± 4.60	53.57 ± 6.19	11.90 ± 4.48	13.85 ± 4.27 11.90 ± 4.48	SHS	A + B/A	• CT: 1 session/day, 30 min. • Motor imagery therapy: 2 sessions/day. • Total course: 25 days. • Per session (MGI): 9–12 min (2–3 min relaxation + 5–7 min imagery + 2 min withdrawal).	① ③ ⑤ ⑧
Shen. Z 2024 (56)	40/40	30/10	27/13	68.03 ± 5.75	66.53 ± 6.58	——	——	SHS	A + C/A	• Basic treatment: ongoing. • Electrical stimulation (ES): 1 session/day, 6 days/week, 20 min/session. • Visual motor imagery training: 1 session/day, 6 days/week, 7–12 min/session (2–3 min relaxation + 5–10 min imagery + 2 min relaxation). • Total course: 6 weeks.	③ ⑧
Cao. SD 2022 (57)	63/63	35/28	32/31	56.25 ± 5.84	57.13 ± 5.28	40.51 ± 4.02	41.12 ± 4.13	SHS	A + C/A	• Basic treatment: ongoing. • ES: 1 session/day, 6 days/week, 20 min/session. • Visual motor imagery training: 1 session/day, 6 days/week, 7–12 min/session (2–3 min relaxation + 5–10 min imagery + 2 min relaxation). • Total course: 6 weeks.	③ ⑧
Wang. J 2012 (58)	30/30	18/12	20/10	60.1 ± 4.9	61.3 ± 5.1	45.1 ± 3.2	47.5 ± 4.6	SS	A + B/A	• CT: 1 session/day, 6 days/week. • Motor imagery therapy: 1 session/day, 6 days/week. • Total course: 4 weeks. • Per session (MGI): 20–25 min (2–3 min relaxation + 5–10 min imagery + 2 min attention focus).	③ ⑧
Zhang. YX 2022 (59)	56/56	32/24	37/19	58.99 ± 7.20	58.67 ± 7.17	——	——	PSP	A + B/A	4周、8周 Total course: 4 and 8 weeks. (Specific frequency and session duration not clearly reported).	① ⑦ ⑨
Chen. CX2019^1^ (8)	50/45	16/34	25/20	60.38 ± 10.12	59.56 ± 8.80	——	——	PSP	A + B/A	• During hospitalization: 2 sessions/day (noon and bedtime). • After discharge: 1 session/day (bedtime), 3–5 days/week. • Course: 2 weeks (in-hospital) + 4 weeks (home) = 6 weeks total. • Per session: 20–30 min.	① ⑦
Jiang. H 2019^1^ (38)	50/45	20/30	25/20	60.38 ± 10.12	59.56 ± 8.80	——	——	PSP	A + B/A	• During hospitalization: 2 sessions/day (noon and bedtime). • After discharge: 1 session/day (bedtime), 3–5 days/week. • Course: 1 week (in-hospital) + 4 weeks (home) = 5 weeks total. • Per session: 20–30 min.	①
Guan. F 2020^1^ (39)	50/50	20/30	25/20	60.38 ± 10.12	59.56 ± 8.80	——	——	PSP	A + B/A	• During hospitalization: 2 sessions/day (noon and bedtime). • After discharge: 1 session/day (bedtime), 3–5 days/week. • Per session: 20–30 min. (Total course not explicitly stated, inferred as ongoing during study period).	① ⑩
Liu. X 2021 (60)	40/40	24/16	22/18	56.71 ± 3.23	55.18 ± 2.24	——	——	PSP	A + B/A	• During hospitalization: 2 sessions/day (morning and evening). • After discharge: 1 session/day, 4–5 days/week. • Course: 1 week (in-hospital) + 4 weeks (home) = 5 weeks total. • Per session: 20 min (in-hospital) / 30 min (home).	① ⑩
Zhou. LL 2022 (61)	50/50	23/27	22/28	63.22 ± 4.26	63.09 ± 4.21	——	——	PSP	A + B/A	• During hospitalization: 2 sessions/day (noon and bedtime). • After discharge: 1 session/day (bedtime), 3–5 days/week. • Total course: 3 weeks. • Per session: 20–30 min (audio-guided multi-sensory scene imagery).	① ⑦ ⑪ ⑫

Data are presented as mean ± SD or as indicated. T, Experimental group (intervention group); C, Control group; CT, Conventional therapy; MGI, Motor Guided Imagery therapy; EA, Electroacupuncture; TENS, Transcutaneous Electrical Nerve Stimulation; ES, Electrical Stimulation; SHS, shoulder subluxation; SS, Shoulder-Hand Syndrome (Complex Regional Pain Syndrome type I); PSP, Post-Stroke Pain (unspecified or mixed type). Interventions are coded as: A, Conventional therapy; B, MGI therapy alone; C, MGI combined with a specific adjunctive therapy. 1, These publications are derived reports from one single parent trial with overlapping participants. Assessment Tools (numerical codes): ① Visual Analogue Scale (VAS); ② Numeric Rating Scale (NRS); ③ Fugl-Meyer Assessment for Upper Extremity (FMA-UE); ④ Shoulder Range of Motion (ROM); ⑤ Degree of limb swelling; ⑥ Activities of Daily Living (ADL) scale; ⑦ Pittsburgh Sleep Quality Index (PSQI); ⑧ Modified Barthel Index (MBI); ⑨ Generic Quality of Life Inventory-74 (GQOLI-74); ⑩ Hospital Anxiety and Depression Scale (HADS); ⑪ Hamilton Depression Rating Scale (HAMD); ⑫ Hamilton Anxiety Rating Scale (HAMA). Data not reported are indicated by “—”.

### 3.2 Risk of bias assessment

Among the 13 included studies, all were judged as having some concerns for the overall risk of bias. For individual domains: all 13 studies were rated as low risk in the randomization process domain (D1); three studies were low risk and the remaining 10 had some concerns in the domain of deviations from the intended interventions (D2); all 13 studies were low risk for missing outcome data (D3); two studies were low risk and the remaining 11 had some concerns for measurement of the outcome (D4); all 13 studies had some concerns for selection of the reported result (D5). The overall risk of bias across included studies was dominated by some concerns (Figure 2).

**Figure 2:**
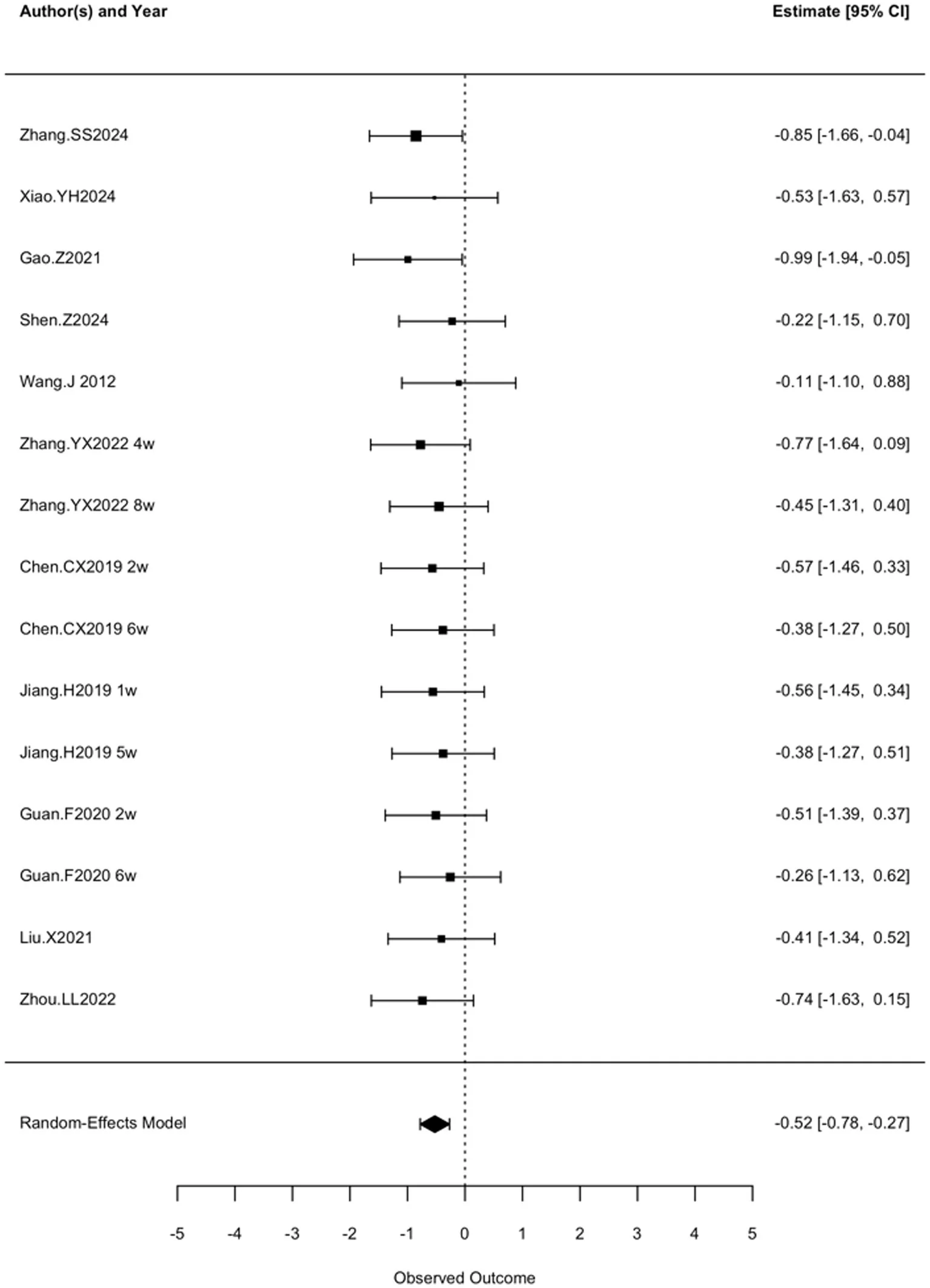
Risk of bias assessment for included studies.

### 3.3 Main effect analysis

A three-level random-effects model (rma.mv) was used to analyze the overall effect of motor-guided imagery (MGI) on pain outcomes in stroke patients, with verification using a two-level random-effects model (rma.uni). Effect sizes were expressed as Hedges’ g. A negative effect size indicates a reduction in pain scores, representing improvement in the intervention group.

#### 3.3.1 Main effect of MGI on pain outcomes in stroke patients

##### 3.3.1.1 Overall effect analysis

A three-level random-effects model was used to analyze the effect of motor-guided imagery therapy on pain outcomes in stroke patients based on 11 reports originating from 9 included RCTs. The pooled effect size was *g* = −0.5240 (95% CI: −0.7788 to −0.2691, *p* = 0.0006), indicating a significant medium effect. The two-level random-effects model confirmed identical results (*g* = −0.5240, 95% CI: −0.7788 to −0.2691, *p* = 0.0006), supporting that MGI significantly reduces pain severity in stroke patients (Figure 3).

**Figure 3:**
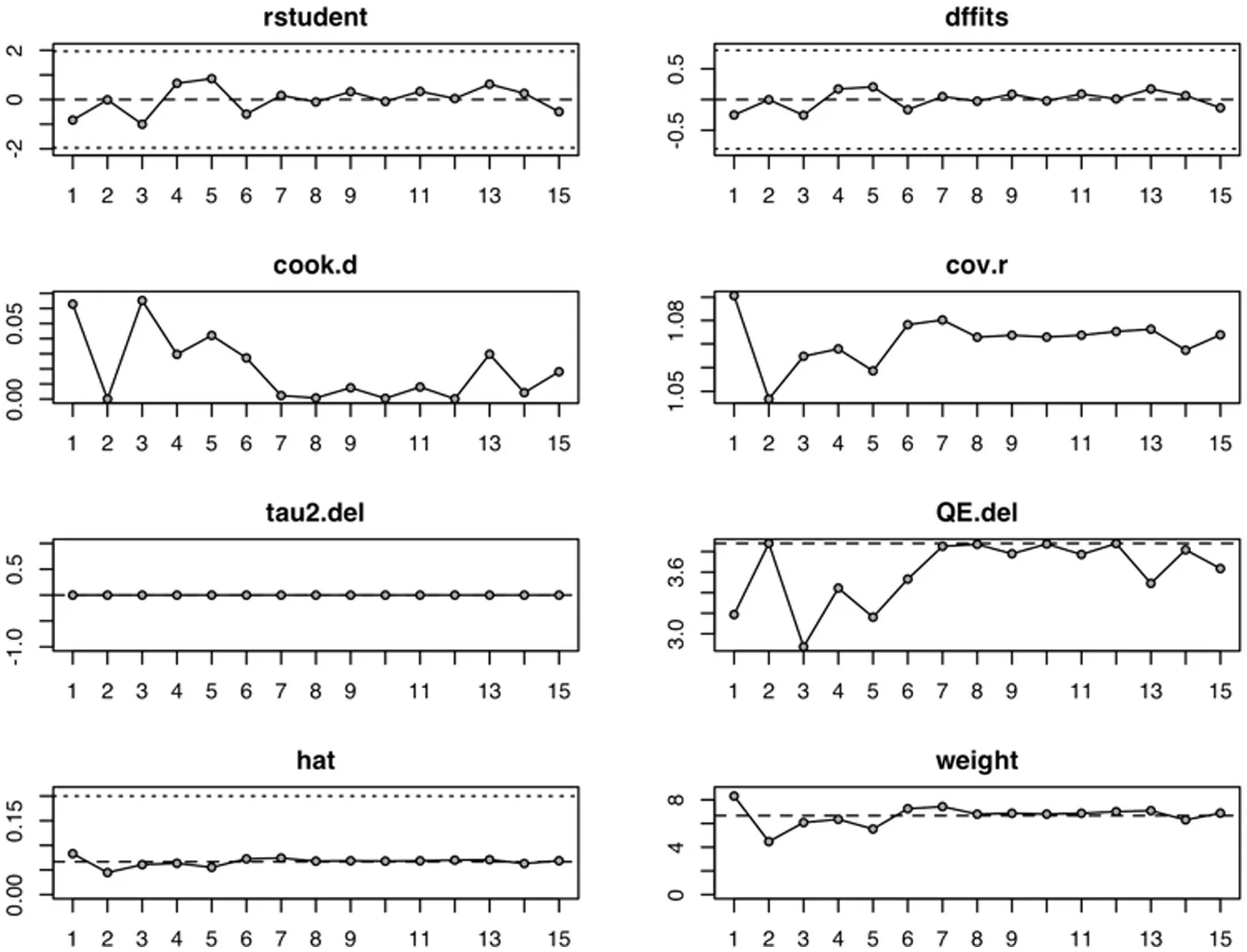
Forest plot for pain outcomes.

##### 3.3.1.2 Influence analysis

Influence analysis was performed using the leave-one-out method. After sequentially removing each effect size and recalculating the pooled effect, the pain outcome effect size ranged from −0.4942 to −0.5483, with all values remaining statistically significant (*p* < 0.05). Heterogeneity remained unchanged (*I*^2^ = 0.00% in all models), and tau^2^ was consistently 0.0000. No highly influential outliers were detected, confirming that the main effect was stable and not driven by any single study or effect size (Figure 4).

**Figure 4:**
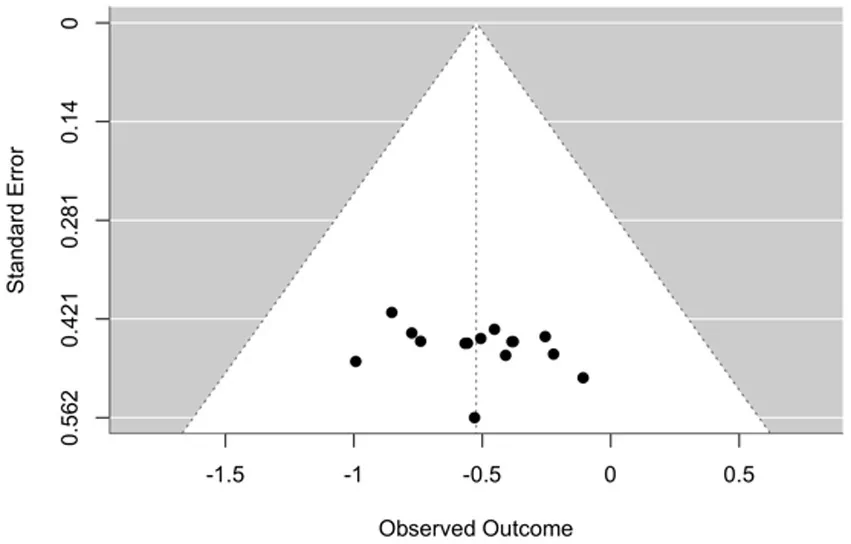
Influence analysis plot for pain outcomes.

To evaluate the contribution of effect sizes originating from the overlapping parent-trial reports of Chen et al. (8), Jiang et al. (38), and Guan et al. (39), an additional sensitivity analysis was conducted by removing all effect sizes from these three publications and refitting the three-level meta-analysis. Before removal, the pooled effect was *g* = −0.524 (95% CI: −0.778 to −0.269, *p* < 0.001). After removing effect sizes from the overlapping sample, the pooled effect was g = −0.583 (95% CI: −0.941 to −0.225, *p* = 0.006). The direction of effect remained unchanged, the confidence interval did not cross the null value, and the result remained statistically significant, suggesting that the pooled estimate for pain outcomes was relatively robust. Nevertheless, the standard error increased and the *p*-value became larger, indicating that this dataset contributed statistical power to the analysis. The test for between-study heterogeneity after removal yielded *Q* = 3.16 (*p* = 0.924, *I*^2^ = 0.00%), showing no detectable between-study heterogeneity (Figure 5).

**Figure 5:**
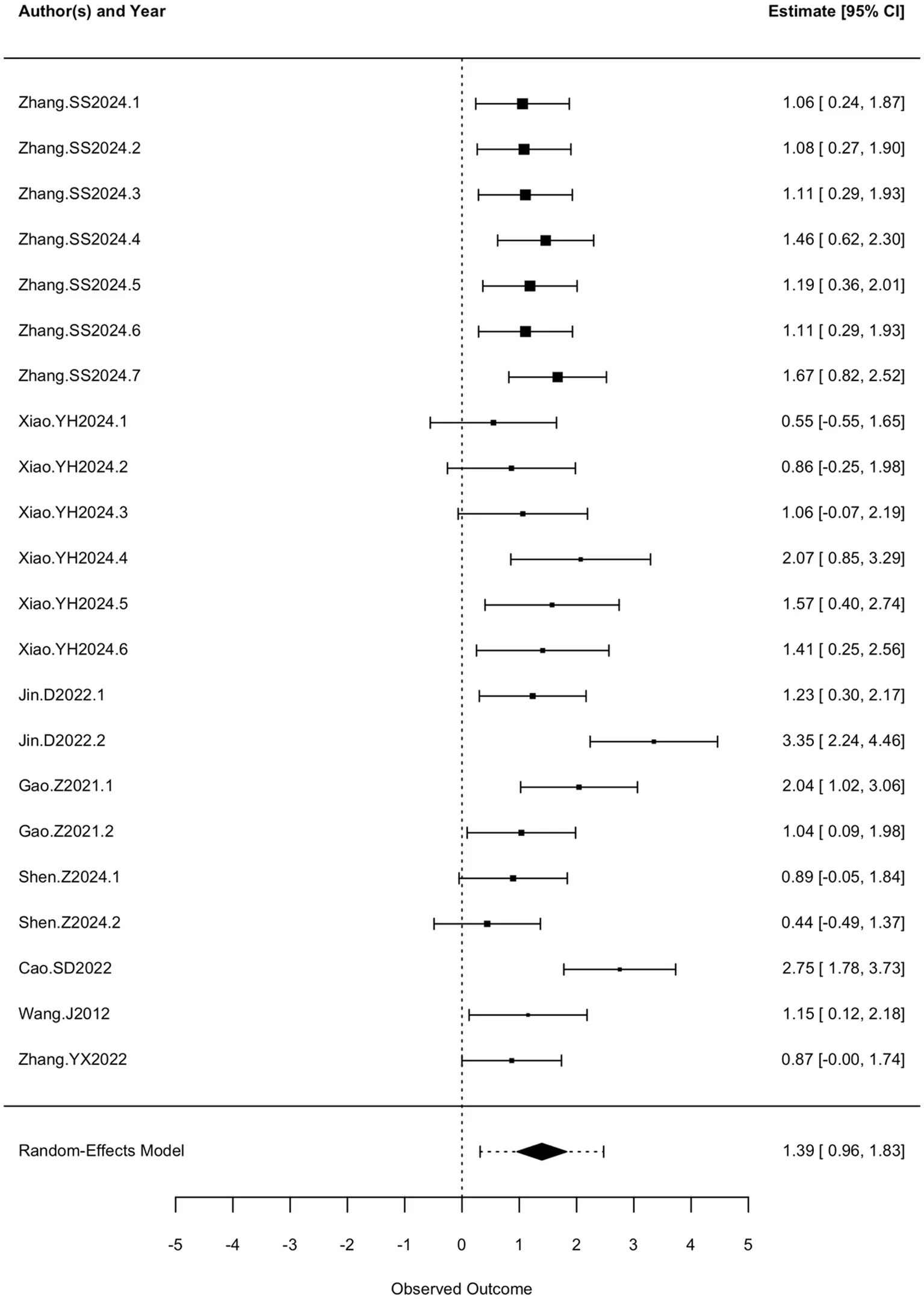
Forest plot of three-level meta-analysis after removing effect sizes from the overlapping parent-trial reports.

##### 3.3.1.3 Heterogeneity analysis

The three-level model heterogeneity test yielded an overall Q statistic of 3.8803 (df = 14, *p* = 0.9961 > 0.05), with an *I*^2^ of 0.00%, indicating no detectable statistical heterogeneity within the current sample. Variance decomposition showed that both within-study variance (Level 2) and between-study variance (Level 3) were 0.0000. A total of 100% of the total variance was attributable to sampling error, while the proportions of within-study and between-study random variation approached zero. Likelihood-ratio tests further confirmed that neither of the two random-effect variance components reached statistical significance (*p* = 1.0000).

Importantly, the zero-heterogeneity point estimate observed in the present dataset does not rule out potential true between-study or within-study variability. Given the limited number of included studies, the heterogeneity estimate itself carries statistical uncertainty and may be underpowered to detect subtle variation. Despite the negligible estimated heterogeneity, the three-level model was retained to appropriately account for the nested structure of multiple effect sizes within single studies, ensuring rigorous methodological specification for dependent data.

##### 3.3.1.4 Moderator analyses

A three-level mixed-effects model was employed to examine the effects of potential moderating variables. The results are as follows (see Table 3).

**Table 3:** Multiple regression analysis for moderator variables.

Moderator variable	*k*	g (95% confidence interval)	*P-*value	Test for moderating effect
Intervention type	15			
Combined Therapy	5	−0.587 (−1.029,-0.146)	0.013	*F*(1, 13) = 0.145, *p* = 0.710
MGI Alone	10	−0.492 (−0.807,-0.176)	0.005	
Age (years)	15			
≤ 60	12	−0.524 (−0.834,-0.213)	0.003	*F*(1,13) = 0.000, *p*-val = 0.996
> 60	5	−0.525 (−0.982,-0.068)	0.028	
Single session duration (min)	15			
Short: ≤30 min	7	−0.512(−0.897,-0.127)	0.013	*F*(df1 = 2, df2 = 12) = 0.047, *p*-val = 0.954
Long: >30 min	6	−0.505(−0.914,-0.097)	0.020	
unclear	2	−0.612(−1.288,-0.064)	0.072	
Disease stage	15			
Acute ≤ 4 weeks	2	−0.912(−1.594,-0.230)	0.013	*F*(2,12) = 1.156, *p*-val = 0.347
Subacute > 4 weeks	3	−0.268(−0.908,0.372)	0.380	
unclear	10	−0.504(−0.815, −0.193)	0.004	
Female proportion (%)	15			
≥ 50%	5	−0.487(−0.923,-0.050)	0.031	*F*(1, 13) = 0.053, *p*-val = 0.822
< 50%	10	−0.544(−0.861,-0.226)	0.003	
Frequency (days/week)	15			
≤ 5 days	10	−0.525 (−0.84,1–0.210)	0.004	*F*(2, 12) = 0.077, *p*-val = 0.926
> 5 days	3	−0.448 (−1.059,0.163)	0.136	
unclear	2	−0.612(−1.288,0.064)	0.072	
Intervention course	15			
Short: ≤4 weeks	6	−0.604(−1.024,-0.184)	0.008	*F*(1,13) = 0.269, *p*-val = 0.613
Long: >4 weeks	9	−0.476(−0.801,-0.152)	0.007	
Frequency (sessions/week)	15			
Low: ≤5	5	−0.380(−0.841,0.080)	0.097	*F*(2,12) = 0.341, *p*-val = 0.718
High: >5	8	−0.585(−0.938,-0.231)	0.004	
unclear	2	−0.612(−1.288,0.064)	0.072	
Total exposure (minutes)	15			
Low Dose: ≤ 360	4	−0.541(−1.058,-0.024)	0.042	*F*(2, 12) = 0.060, *p*-val = 0.942
High Dose: >360 min	9	−0.495(−0.829,-0.162)	0.007	
unclear	2	−0.612(−1.288,0.064)	0.072	
Pain location	15			
Shoulder	5	−0.569(−1.032,-0.107)	0.020	*F*(1, 13) = 0.065, *p*-val = 0.803
Mixed	10	−0.504(−0.812,-0.195)	0.004	

Three-level mixed-effects models were used to examine potential moderators, and the results are presented below:

1. Intervention type: The moderating effect was not significant (*F* = 0.145, df = 1, 13, *p* = 0.710). Both combined intervention (*g* = −0.587, 95% CI: −1.029 to −0.146, *p* = 0.013) and single intervention (*g* = −0.492, 95% CI: −0.807 to −0.176, *p* = 0.005) yielded significant moderate effects with similar magnitudes.
2. Age: The moderating effect was not significant (*F* = 0.000, df = 1, 13, *p* = 0.996). Both the ≤60 years group (*g* = −0.524, 95% CI: −0.834 to −0.213, *p* = 0.003) and the >60 years group (*g* = −0.525, 95% CI: −0.982 to −0.068, *p* = 0.028) showed significant moderate effects with highly consistent magnitudes.
3. Single intervention duration: The moderating effect was not significant (*F* = 0.047, df = 2, 12, *p* = 0.954). Both the ≤30 min group (*g* = −0.512, 95% CI: −0.897 to −0.127, *p* = 0.013) and the >30 min group (*g* = −0.505, 95% CI: −0.914 to −0.097, *p* = 0.020) were significant with similar effects. The unclear duration group approached significance (*g* = −0.612, 95% CI: −1.288 to −0.064, *p* = 0.072).
4. Disease stage: The moderating effect was not significant (*F* = 1.156, df = 2, 12, *p* = 0.347). Only the acute stage (≤4 weeks; *g* = −0.912, 95% CI: −1.594 to −0.230, *p* = 0.013, large effect) and the unclear stage group (*g* = −0.504, 95% CI: −0.815 to −0.193, *p* = 0.004) were significant, with the acute stage showing the largest effect. The subacute stage (>4 weeks) was not significant (*g* = −0.268, 95% CI: −0.908 to 0.372, *p* = 0.380).
5. Female proportion: The moderating effect was not significant (*F* = 0.053, df = 1, 13, *p* = 0.822). Both the ≥50% female group (*g* = −0.487, 95% CI: −0.923 to −0.050, *p* = 0.031) and the <50% female group (*g* = −0.544, 95% CI: −0.861 to −0.226, *p* = 0.003) showed significant moderate effects with similar magnitudes.
6. Intervention frequency (days/week): The moderating effect was not significant (*F* = 0.077, df = 2, 12, *p* = 0.926). The ≤5 times/week group was significant (*g* = −0.525, 95% CI: −0.841 to −0.210, *p* = 0.004). The >5 times/week group was not significant (*g* = −0.448, 95% CI: −1.059 to 0.163, *p* = 0.136). The unclear frequency group approached significance (*g* = −0.612, 95% CI: −1.288 to 0.064, *p* = 0.072).
7. Intervention period: The moderating effect was not significant (*F* = 0.269, df = 1, 13, *p* = 0.613). Both the ≤4 weeks group (*g* = −0.604, 95% CI: −1.024 to −0.184, *p* = 0.008) and the >4 weeks group (*g* = −0.476, 95% CI: −0.801 to −0.152, *p* = 0.007) showed significant moderate effects, with a slightly larger effect in the shorter period group.
8. Intervention sessions (times/week): The moderating effect was not significant (*F* = 0.341, df = 2, 12, *p* = 0.718). The >5 times/week group was significant (*g* = −0.585, 95% CI: −0.938 to −0.231, *p* = 0.004). The ≤5 times/week group was not significant (*g* = −0.380, 95% CI: −0.841 to 0.080, *p* = 0.097). The unclear sessions group approached significance (*g* = −0.612, 95% CI: −1.288 to 0.064, *p* = 0.072).
9. Total exposure duration: The moderating effect was not significant (*F* = 0.060, df = 2, 12, *p* = 0.942). Both the low-dose group (≤360 min; *g* = −0.541, 95% CI: −1.058 to −0.024, *p* = 0.042) and the medium-dose group (>360 min; *g* = −0.495, 95% CI: −0.829 to −0.162, *p* = 0.007) were significant. The high-dose group did not reach significance.
10. Pain location: The moderating effect was not significant (*F* = 0.065, df = 1, 13, *p* = 0.803). Both shoulder pain (*g* = −0.569, 95% CI: −1.032 to −0.107, *p* = 0.020) and widespread/mixed pain (*g* = −0.504, 95% CI: −0.812 to −0.195, *p* = 0.004) were significant, with minimal differences in effect size.

##### 3.3.1.5 Multiple moderator model

Since no moderator variable showed statistically significant effects (all *p* > 0.05), multiple moderator regression analysis was not performed.

##### 3.3.1.6 Publication bias assessment

Funnel plots, Egger’s regression test, and the trim-and-fill method were used to evaluate potential publication bias. The funnel plot revealed a generally symmetric distribution of effect sizes. Egger’s regression test showed no significant funnel plot asymmetry (*t* = 0.5428, df = 13, *p* = 0.5965). The trim-and-fill method estimated 1 missing study on the left side; after adjustment, the pooled effect size (*g* = −0.5483, 95% CI: −0.7750 to −0.3217) was highly consistent with the original result (*g* = −0.5240, 95% CI: −0.7788 to −0.2691). Nevertheless, with 11 included studies for this outcome, the statistical power of Egger’s regression and trim-and-fill remains suboptimal. Non-significant test results and consistent estimates before and after trim-and-fill adjustment cannot rule out underlying publication-bias risk (Figure 6).

**Figure 6:**
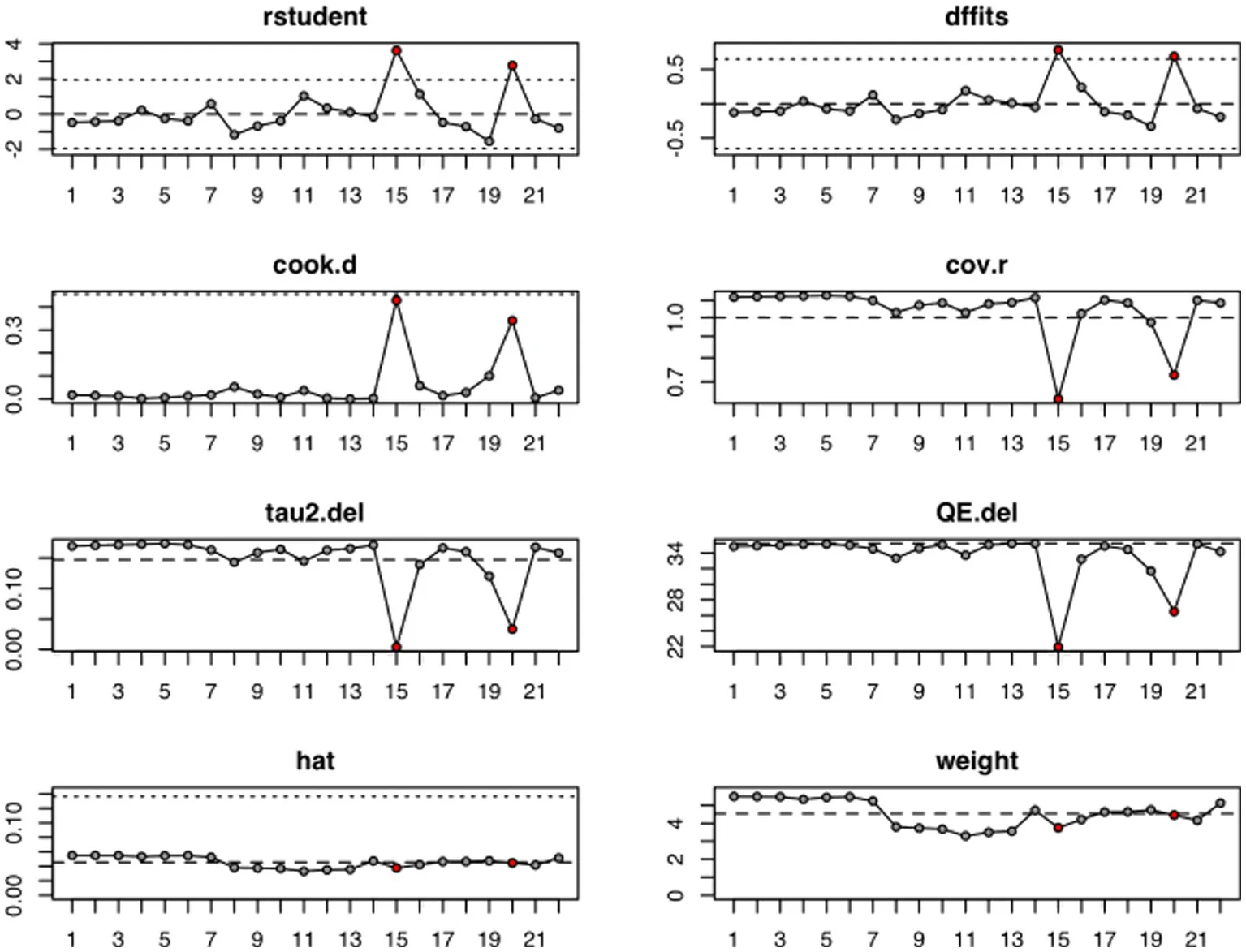
Funnel plot for pain outcomes.

#### 3.3.2 Main effect of MGI on functional outcomes in stroke patients

##### 3.3.2.1 Overall effect analysis

Before removing outliers, the three-level random-effects model showed an effect size of *g* = 1.3949 (95% CI: 0.9634 to 1.8264, *p* < 0.001), representing a large effect for MGI on functional outcomes in stroke patients. Verification with the two-level model yielded *g* = 1.3310 (95% CI: 1.0549 to 1.6071, *p* < 0.001), indicating that MGI significantly improves functional status (Figure 7).

**Figure 7:**
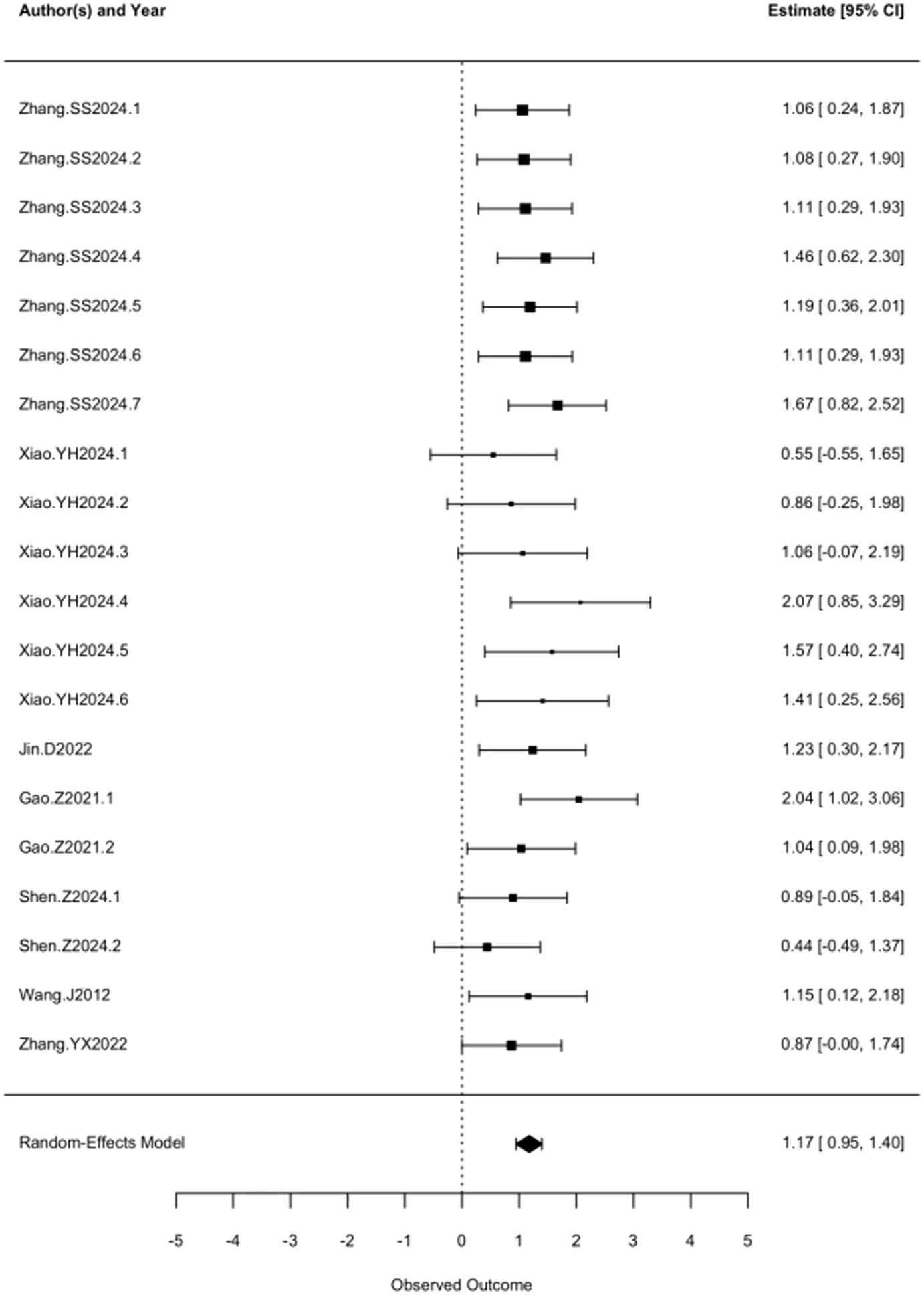
Forest plot of the primary main analysis for functional outcomes.

##### 3.3.2.2 Influence analysis

Influence analysis using the influence function and the leave-one-out method identified Effect Size ID = 15 and 20 as outliers. After their removal, the overall effect size remained significant and fluctuated within a small range (1.1305 to 1.3731). Leave-one-out analysis showed that after sequentially removing each effect size, all *p*-values remained < 0.001, and heterogeneity did not increase significantly, indicating that aside from the identified outliers, no other study substantially influenced the overall result (Figure 8).

**Figure 8:**
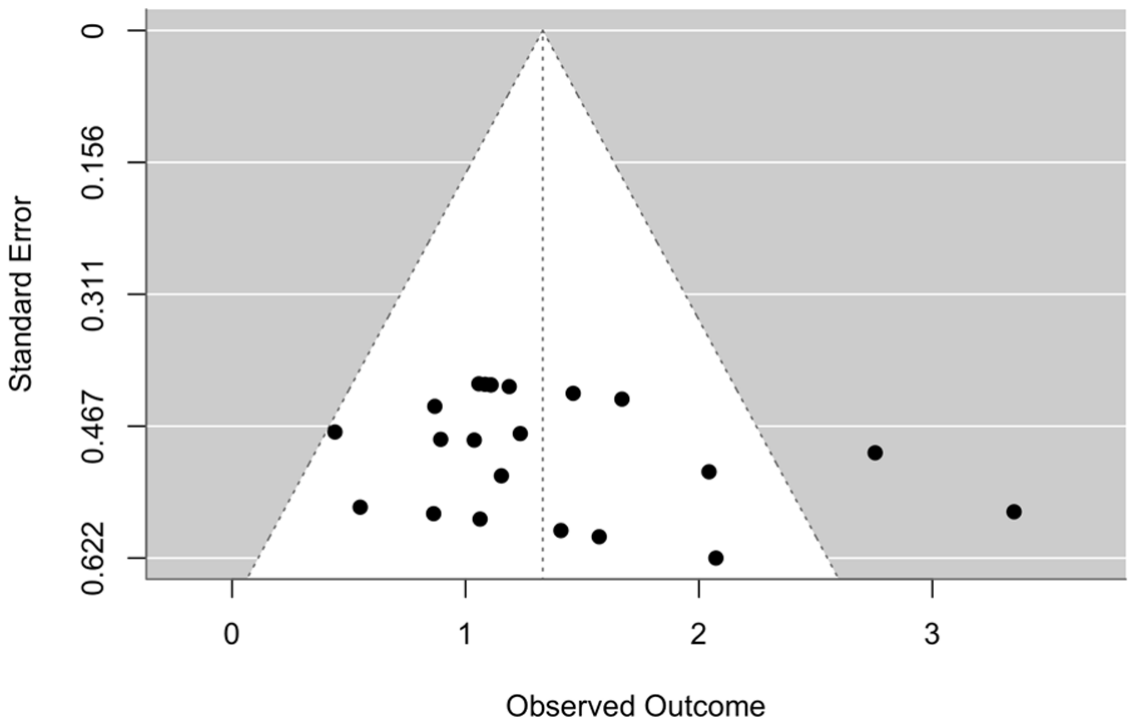
Influence analysis plot for functional outcomes.

After removing outliers (Effect Size ID = 15 and 20, with rstudent values of 3.6255 and 2.7682, and cook.d values of 0.4288 and 0.3407, respectively), the three-level model effect size from this sensitivity analysis was g = 1.1735 (95% CI: 0.9488 to 1.3981, *p* < 0.001), still a large effect. The two-level model result was consistent (*g* = 1.1735, 95% CI: 0.9488 to 1.3981, *p* < 0.001), indicating stable intervention effects (Figure 9).

**Figure 9:**
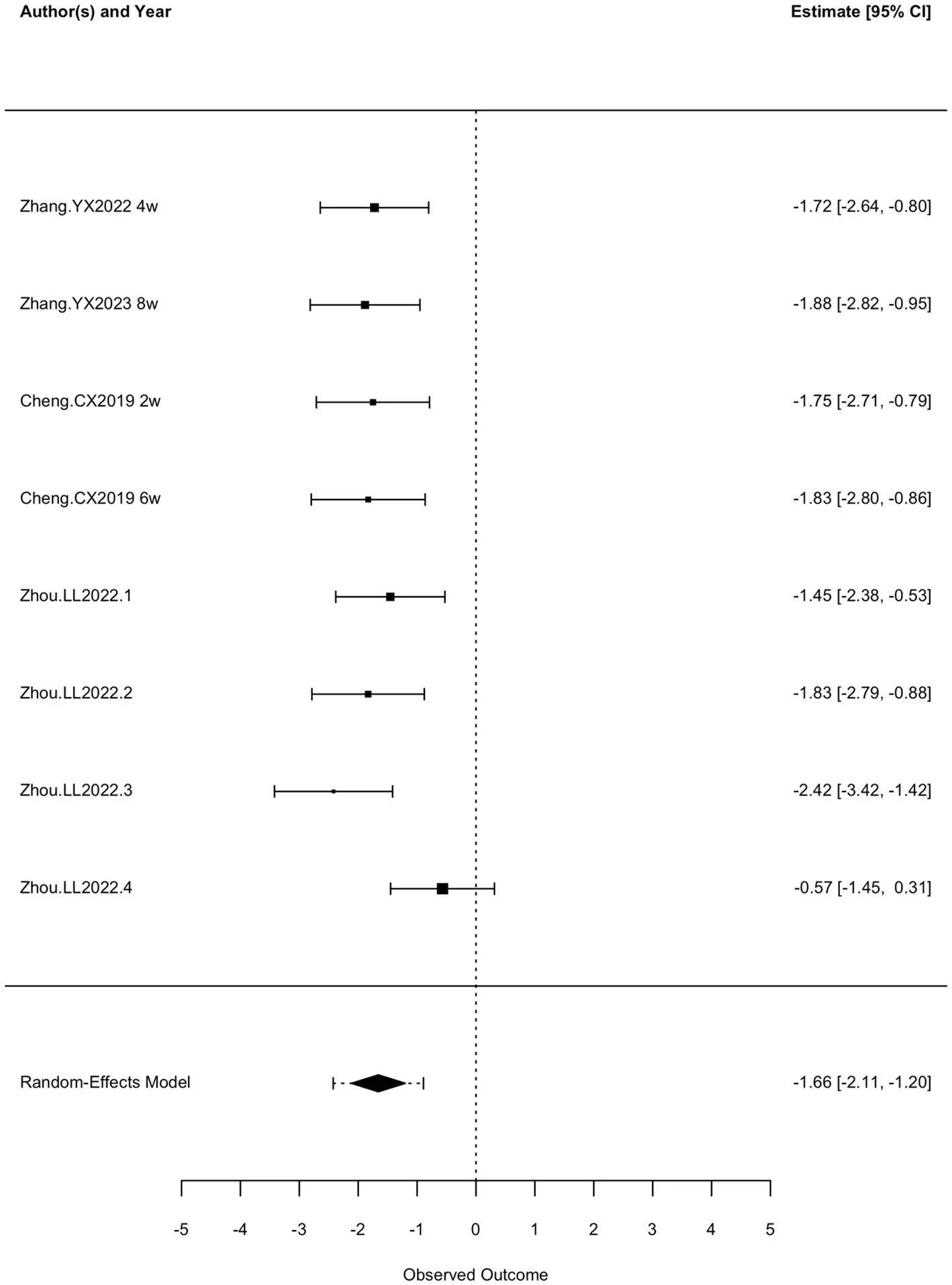
Forest plot of the sensitivity analysis for functional outcomes.

##### 3.3.2.3 Heterogeneity analysis

Before outlier removal, heterogeneity testing indicated *Q* = 35.2487 (df = 21, *p* = 0.0265), *I*^2^ = 38.52%, suggesting moderate heterogeneity. Variance component decomposition showed: Level 2 (within-study) variance σ^2^.1 = 0.0174 (*p* = 0.8805), Level 3 (between-study) variance σ^2^.2 = 0.2084 (*p* < 0.05), indicating between-study variance was the primary source of heterogeneity. Sampling variance accounted for 38.48%, within-study variance for 4.52%, and between-study variance for 57.00% of the total variance. After outlier removal, heterogeneity was eliminated: *Q* = 12.2944 (df = 19, *p* = 0.8727), I^2^ = 0.00%; both Level 2 and Level 3 variances were 0.0000 (*p* = 1.0000), and sampling variance accounted for 100.00%. Log-likelihood ratio tests indicated no significant difference in fit between the three-level and two-level models after outlier removal, but the three-level model was retained to reflect the nested data structure.

##### 3.3.2.4 Moderator analysis

The analysis of all moderating variables (gender, age, intervention characteristics, etc.) showed no significant moderating effects (all *p* > 0.05).

Subgroup analysis stratified by measurement tool was performed to explore the influence of different assessment modalities on the pooled effect of MGI on upper-limb function in patients after stroke. A total of 22 independent effect sizes were included and divided into four subgroups. The pooled effect sizes for each subgroup were as follows: FMA (*g* = 0.99, 95%CI 0.15–1.83, *p* = 0.024); GQOLI-74 physical subscale (*g* = 1.88, 95%CI 0.75–3.02, *p* = 0.003); ROM (range of motion of shoulder joint) (*g* = 1.65, 95%CI 0.71–2.60, *p* = 0.002); swelling volume (*g* = 1.74, 95%CI 0.85–2.62, *p* < 0.001). The test for subgroup differences showed no statistically significant difference (*F* = 1.49, df₁ = 3, df₂ = 18, *p* = 0.251) (see Table 4).

**Table 4:** Multiple regression analysis for moderator variables.

Moderator	k	g (95% CI)	*p*-value	Test of moderator effects
Measurement tool	22			*F*(3,18) = 1.49, *p* = 0.251
FMA	5	0.99 (0.15, 1.83)	0.024	
GQOLI-74 physical subscale	2	1.88 (0.75, 3.02)	0.003	
ROM (range of motion)	11	1.65 (0.71, 2.60)	0.002	
Swelling volume	4	1.74 (0.85, 2.62)	<0.001	

##### 3.3.2.5 Multiple moderator model

Since no significant moderators were identified, multiple moderator regression analysis was not performed.

##### 3.3.2.6 Publication bias assessment

Publication-bias assessment was performed on the primary main-analysis dataset retaining all effect-size estimates, including high-influence outlying points. Egger’s regression test for funnel-plot asymmetry yielded *t* = 1.4385 (df = 20, *p* = 0.1657); the intercept estimate as the standard error approached zero was *b* = −0.0529 (95% CI: −2.0752 to 1.9694). No statistically significant funnel-plot asymmetry was detected (Figure 10).

**Figure 10:**
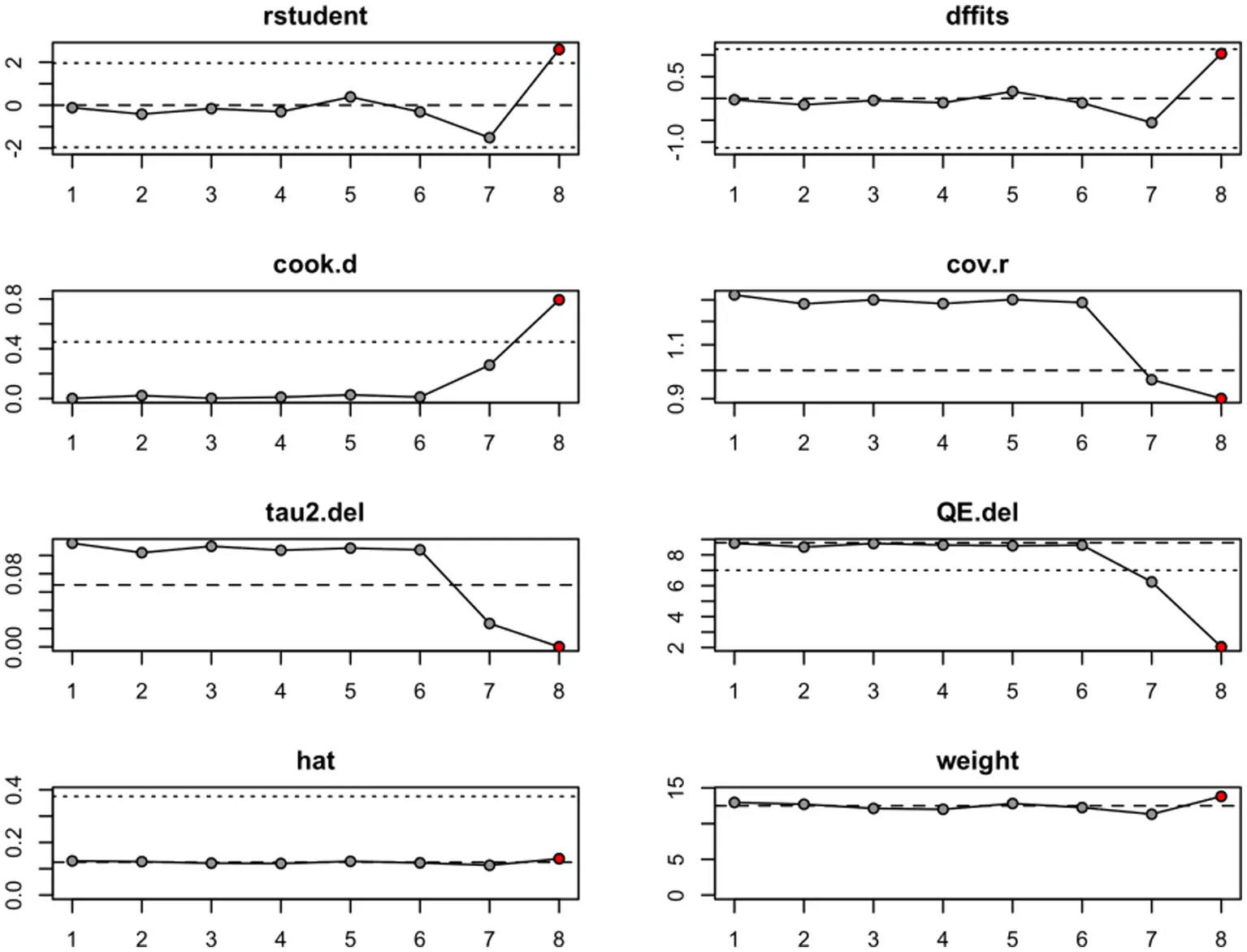
Funnel plot for functional outcomes.

It should be noted that the number of observed effect-size estimates in the present study was limited. Egger’s regression possesses low statistical power under such sample conditions, and potential publication bias cannot be fully excluded based solely on this test result. Publication-bias tests were not repeated for the sensitivity-analysis dataset after removal of high-influence effect-size points.

#### 3.3.3 Main effect of MGI on sleep outcomes in stroke patients

##### 3.3.3.1 Overall effect analysis

Before outlier removal, the three-level random-effects model indicated an effect size of *g* = −1.6578 (95% CI: −2.1147 to −1.2010, *p* < 0.001) for MGI on sleep outcomes in stroke patients, representing a large effect (negative value indicates better sleep outcomes in the intervention group compared to control/baseline). The two-level model verification was consistent (*g* = −1.6578, 95% CI: −2.1147 to −1.2010, *p* < 0.001) (Figure 11).

**Figure 11:**
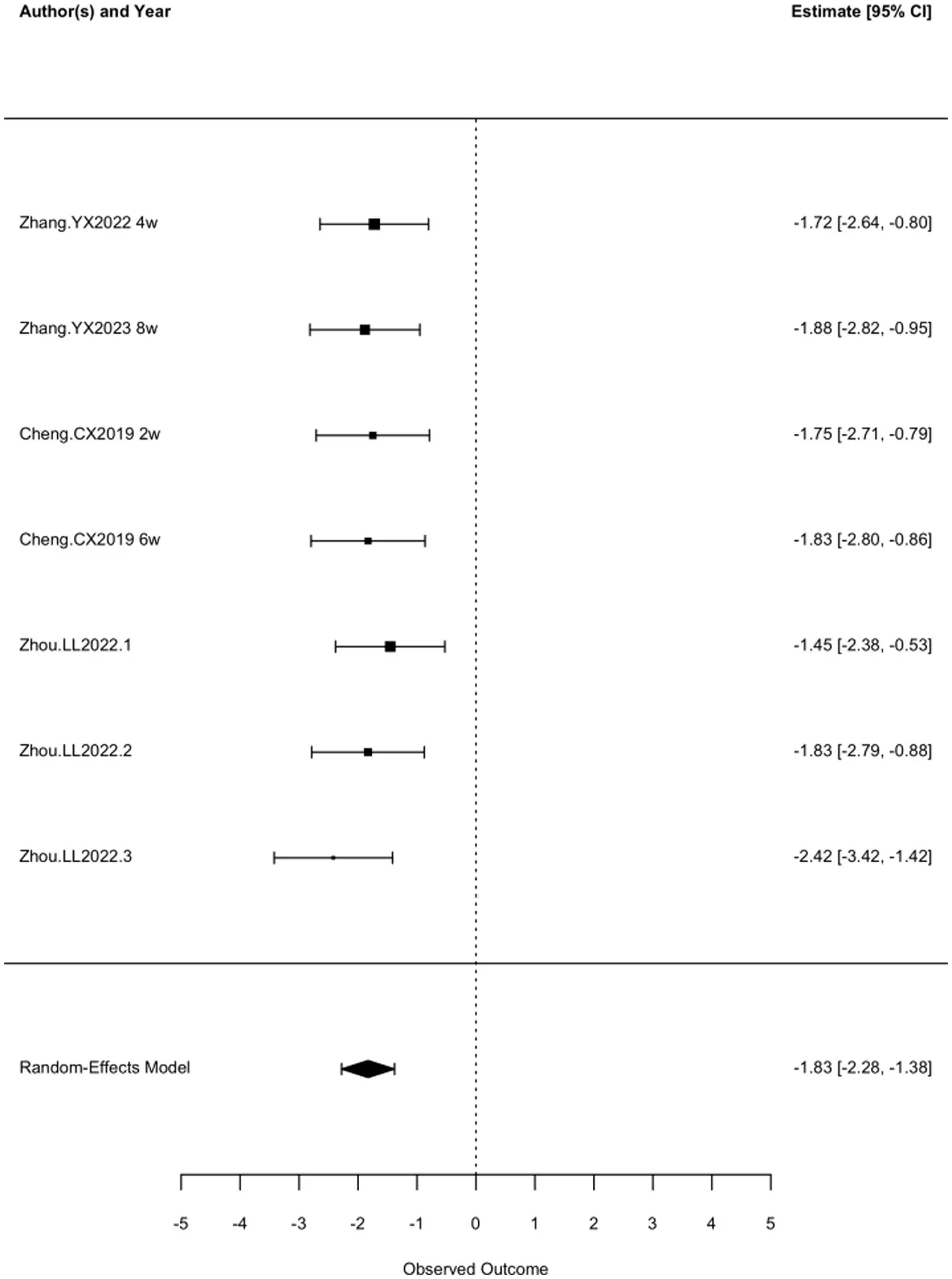
Forest plot of the primary main analysis for sleep outcomes.

##### 3.3.3.2 Influence analysis

The leave-one-out method identified Effect Size ID = 8 as an outlier (removal led to tau^2^ decreasing from 0.0677 to 0.0000 and *I*^2^ from 22.69 to 0.00%). After sequentially removing other effect sizes, the effect size fluctuated between −1.7432 and −1.8963, all *p*-values remained < 0.001, and heterogeneity did not increase significantly, indicating a stable main effect (Figure 12).

**Figure 12:**
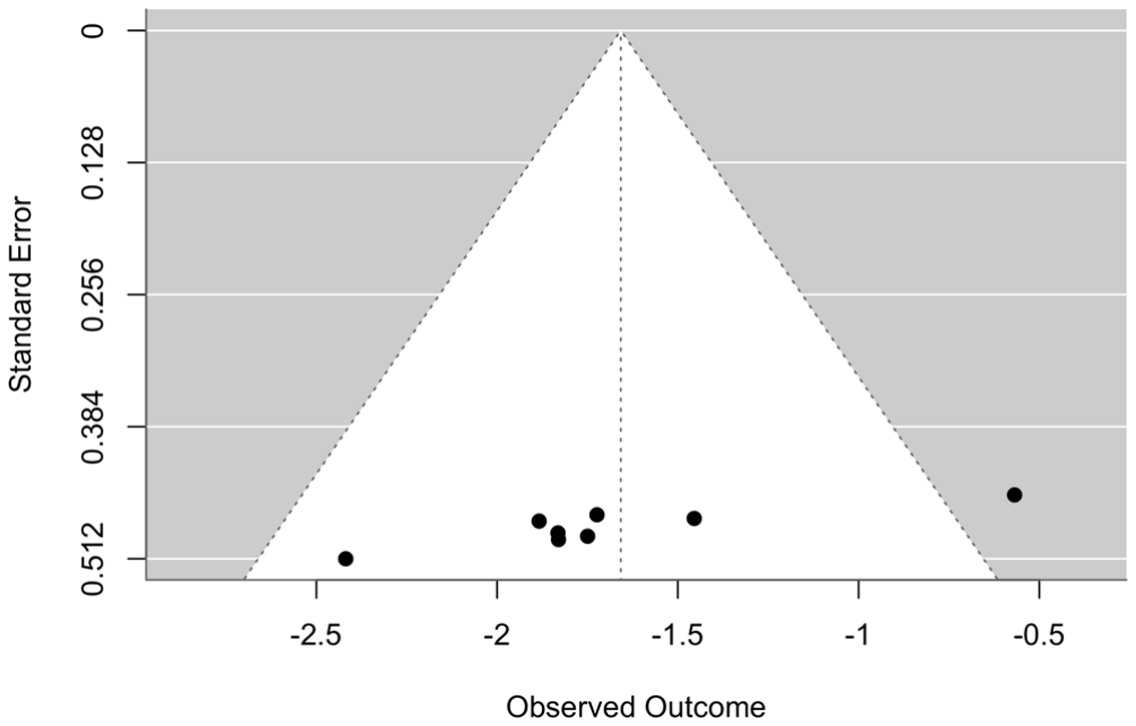
Influence analysis plot for sleep outcomes.

After removing outlier (Effect Size ID = 8), the three-level model effect size from this sensitivity analysis was *g* = −1.8299 (95% CI: −2.2785 to −1.3813, *p* < 0.001), still a large effect with an increased absolute value. The two-level model result was consistent, suggesting the intervention’s effect on sleep outcomes is more pronounced and stable (Figure 13).

**Figure 13:**
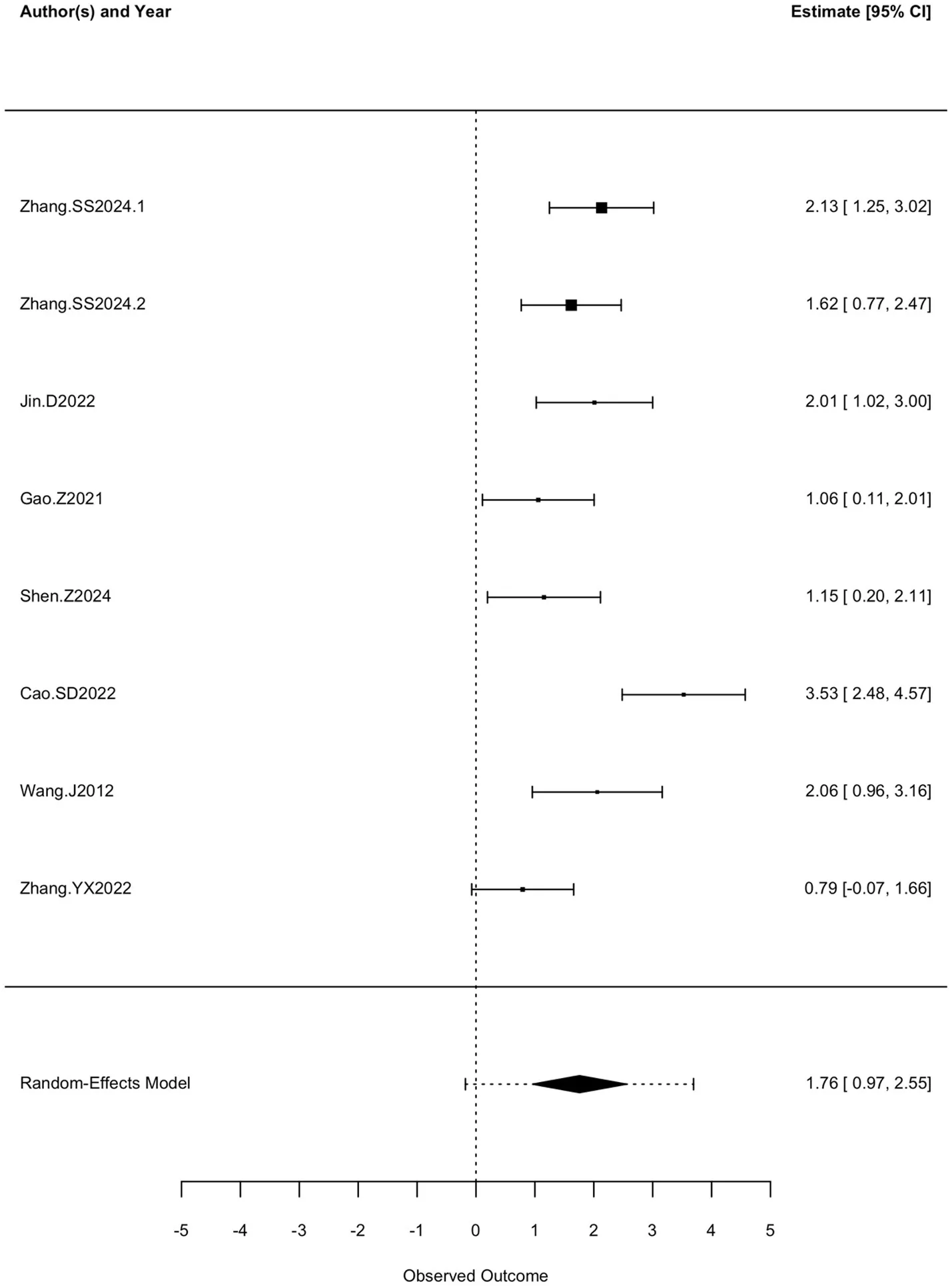
Forest plot of the sensitivity analysis for sleep outcomes.

##### 3.3.3.3 Heterogeneity analysis

Before outlier removal, heterogeneity testing showed *Q* = 8.7838 (df = 7, *p* = 0.2686), *I*^2^ = 22.69%, indicating mild heterogeneity. Variance component decomposition: Level 2 variance σ^2^.1 = 0.0677 (*p* = 0.5949), Level 3 variance σ^2^.2 = 0.0000 (*p* = 1.0000), suggesting within-study variance was the primary source. Sampling variance accounted for 77.31%, within-study variance for 22.69%. After outlier removal, heterogeneity disappeared: *Q* = 2.0448 (df = 6, *p* = 0.9155), *I*^2^ = 0.00%; both Level 2 and Level 3 variances were 0.0000, and sampling variance accounted for 100.00%.

##### 3.3.3.4 Publication bias assessment

Publication-bias assessment was performed on the primary main-analysis dataset retaining all effect-size estimates, including high-influence effect-size points. Egger’s regression test for funnel-plot asymmetry yielded *t* = −2.6430 (df = 6, *p* = 0.0384); the intercept estimate as the standard error approached zero was *b* = 10.7285 (95% CI: −0.7393 to 22.1962). Statistically significant funnel-plot asymmetry was detected (Figure 14).

**Figure 14:**
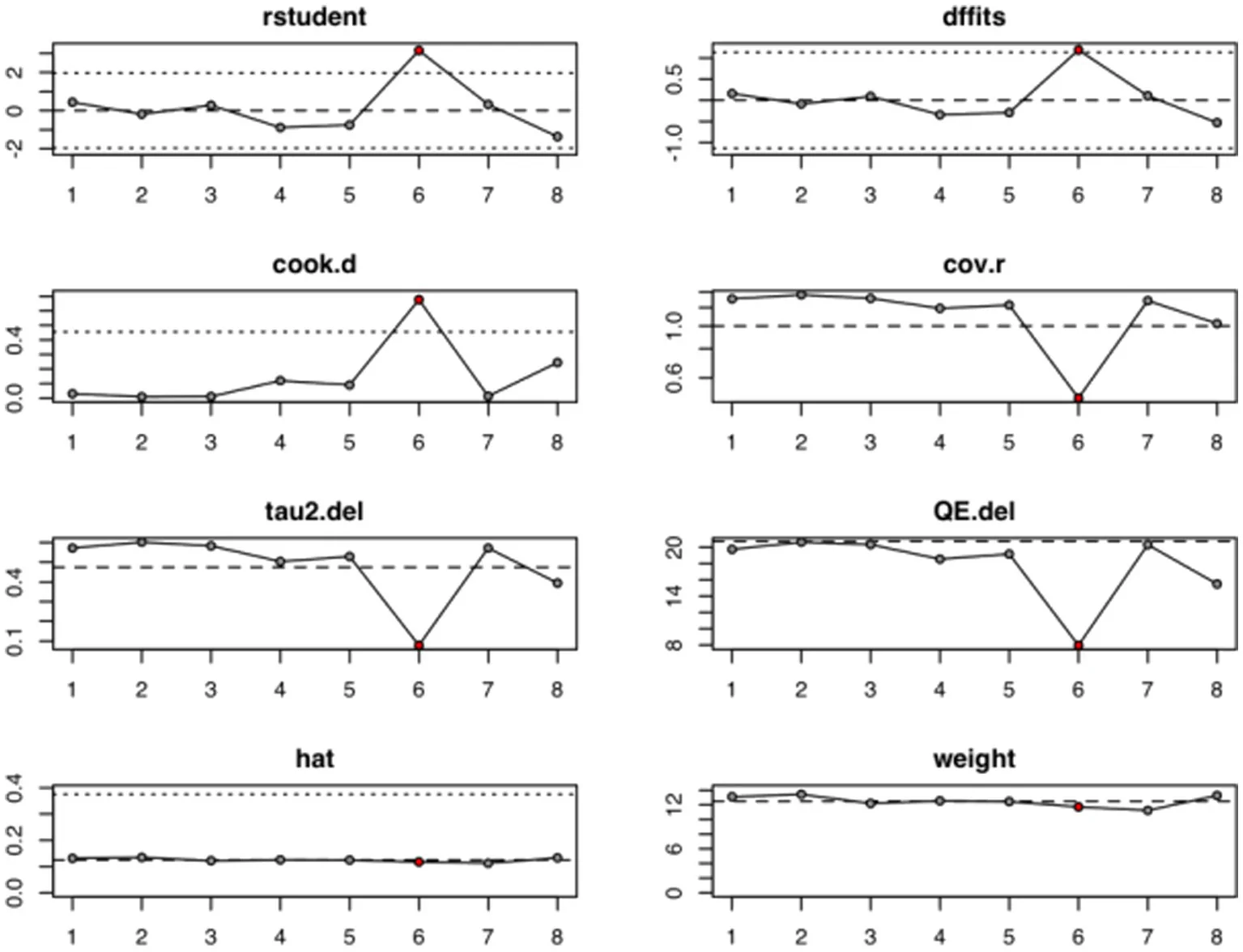
Funnel plot for sleep outcomes.

Caution is warranted when interpreting these publication-bias diagnostics: the number of observed effect-size estimates for sleep outcomes was limited (*k* = 8). Under small-sample conditions, Egger’s regression, visual inspection of funnel plots, and the trim-and-fill method all possess limited statistical power. The significant asymmetry signal detected in this limited dataset cannot be unequivocally attributed to publication bias alone; other factors such as between-study heterogeneity may also contribute to funnel-plot asymmetry. Publication-bias diagnostics were not repeated for the sensitivity-analysis dataset after removal of high-influence effect-size points.

#### 3.3.4 Main effect of MGI on life status outcomes in stroke patients

##### 3.3.4.1 Overall effect analysis

Before removing outliers, the three-level random-effects model showed an effect size of *g* = 1.7572 (95% CI: 0.9656 to 2.5488, *p* = 0.0012) for MGI on life status outcomes in stroke patients, representing a large effect. Verification with the two-level model yielded g = 1.7687 (95% CI: 1.0637 to 2.4737, *p* < 0.001), indicating that MGI significantly improves life status (e.g., quality of life, activities of daily living) (Figure 15).

**Figure 15:**
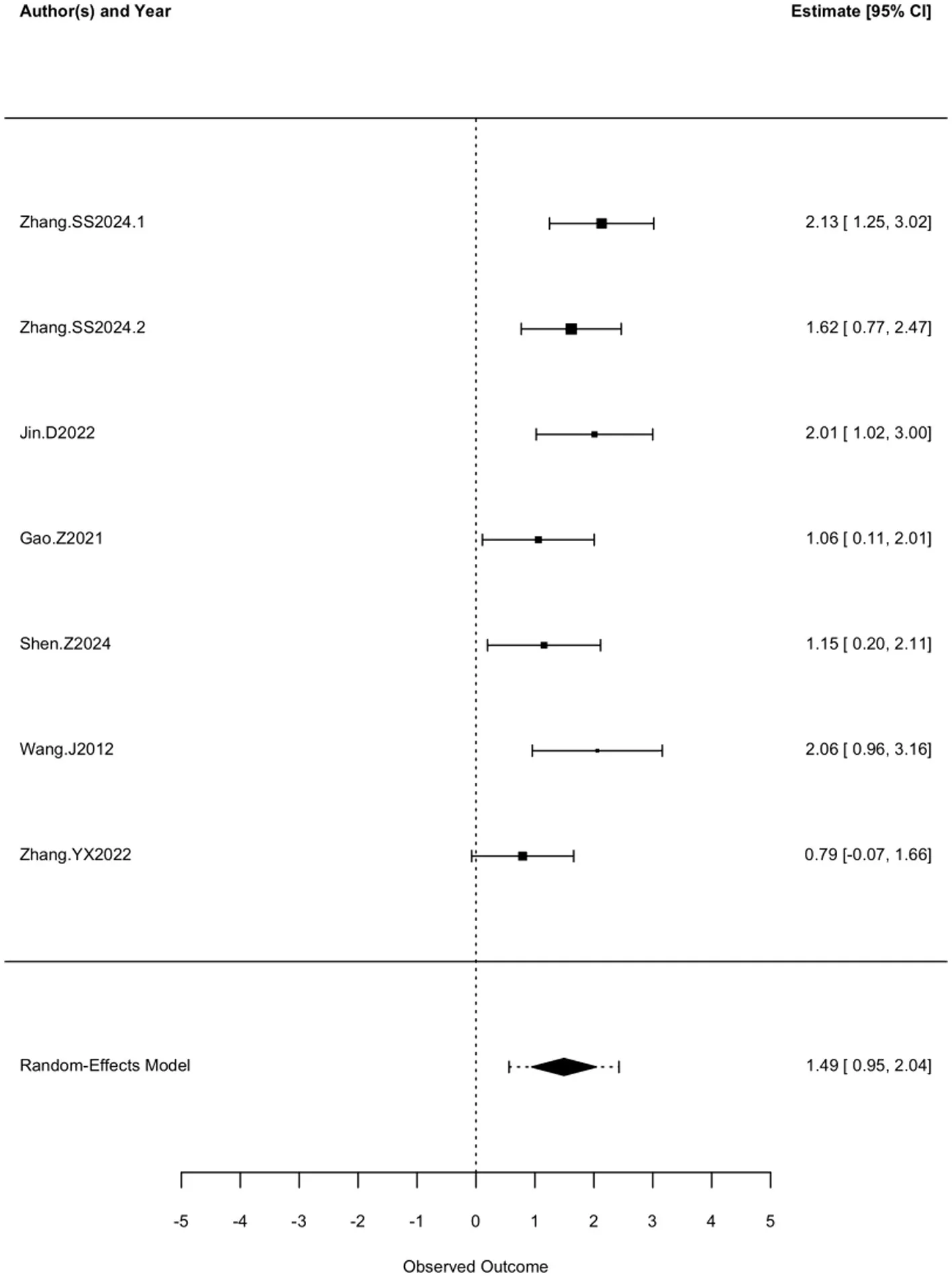
Forest plot of the primary main analysis for life status outcomes.

##### 3.3.4.2 Influence analysis

The leave-one-out method identified Effect Size ID = 6 as an outlier (removal led to tau^2^ decreasing from 0.4742 to 0.0777 and I^2^ from 67.02 to 25.47%). After sequentially removing other effect sizes, the effect size fluctuated between 1.4060 and 1.6615, all *p*-values remained < 0.01, and heterogeneity did not increase significantly, indicating a stable main effect (Figure 16).

**Figure 16:**
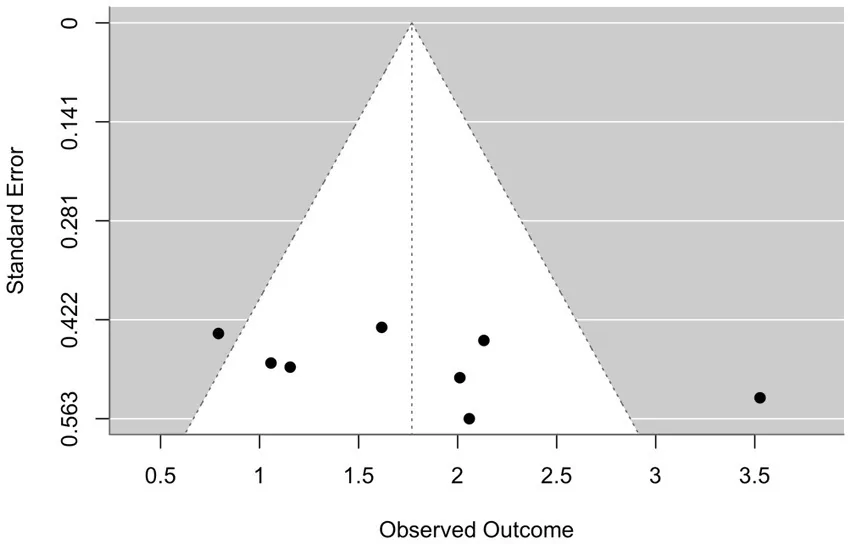
Influence analysis plot for life status outcomes.

After removing outlier (Effect Size ID = 6), the three-level model effect size from this sensitivity analysis was *g* = 1.4944 (95% CI: 0.9467 to 2.0422, *p* = 0.0005), still a large effect. The two-level model result was consistent, suggesting stable intervention effects (Figure 17).

**Figure 17:**
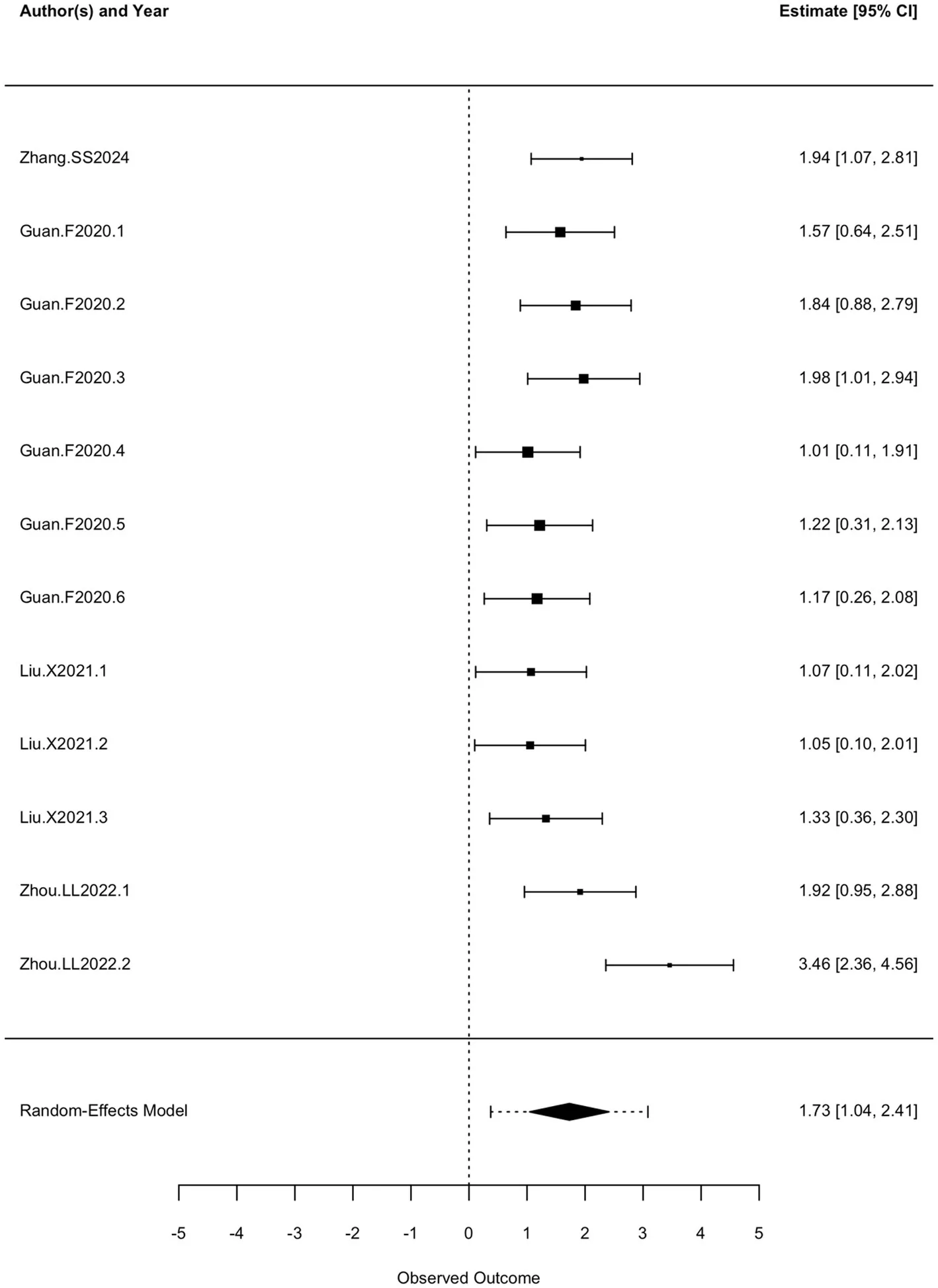
Forest plot of the sensitivity analysis for life status outcomes.

##### 3.3.4.3 Heterogeneity analysis

Before outlier removal, heterogeneity testing showed *Q* = 20.7016 (df = 7, *p* = 0.0042), *I*^2^ = 67.02%, indicating high heterogeneity. Variance component decomposition: Level 2 variance σ^2^.1 = 0.0000 (*p* = 1.0000), Level 3 variance σ^2^.2 = 0.5588 (*p* = 0.5346), suggesting between-study variance was the primary source. Sampling variance accounted for 32.98%, between-study variance for 67.02% of the total variance. After outlier removal, heterogeneity decreased to mild levels: *Q* = 7.9433 (df = 6, *p* = 0.2423), I^2^ = 25.47%; Level 2 variance σ^2^.1 = 0.0000, Level 3 variance σ^2^.2 = 0.0951 (*p* = 0.6604). Sampling variance accounted for 74.53%, and between-study variance for 25.47%.

##### 3.3.4.4 Publication bias assessment

Publication-bias assessment was performed on the primary main-analysis dataset retaining all effect-size estimates, including high-influence observations. Egger’s regression test for funnel-plot asymmetry yielded *t* = 1.5628 (df = 6, *p* = 0.1691); the intercept estimate as the standard error approached zero was *b* = −3.1499 (95% CI: −10.8723 to 4.5724). No statistically significant funnel-plot asymmetry was detected (Figure 18).

**Figure 18:**
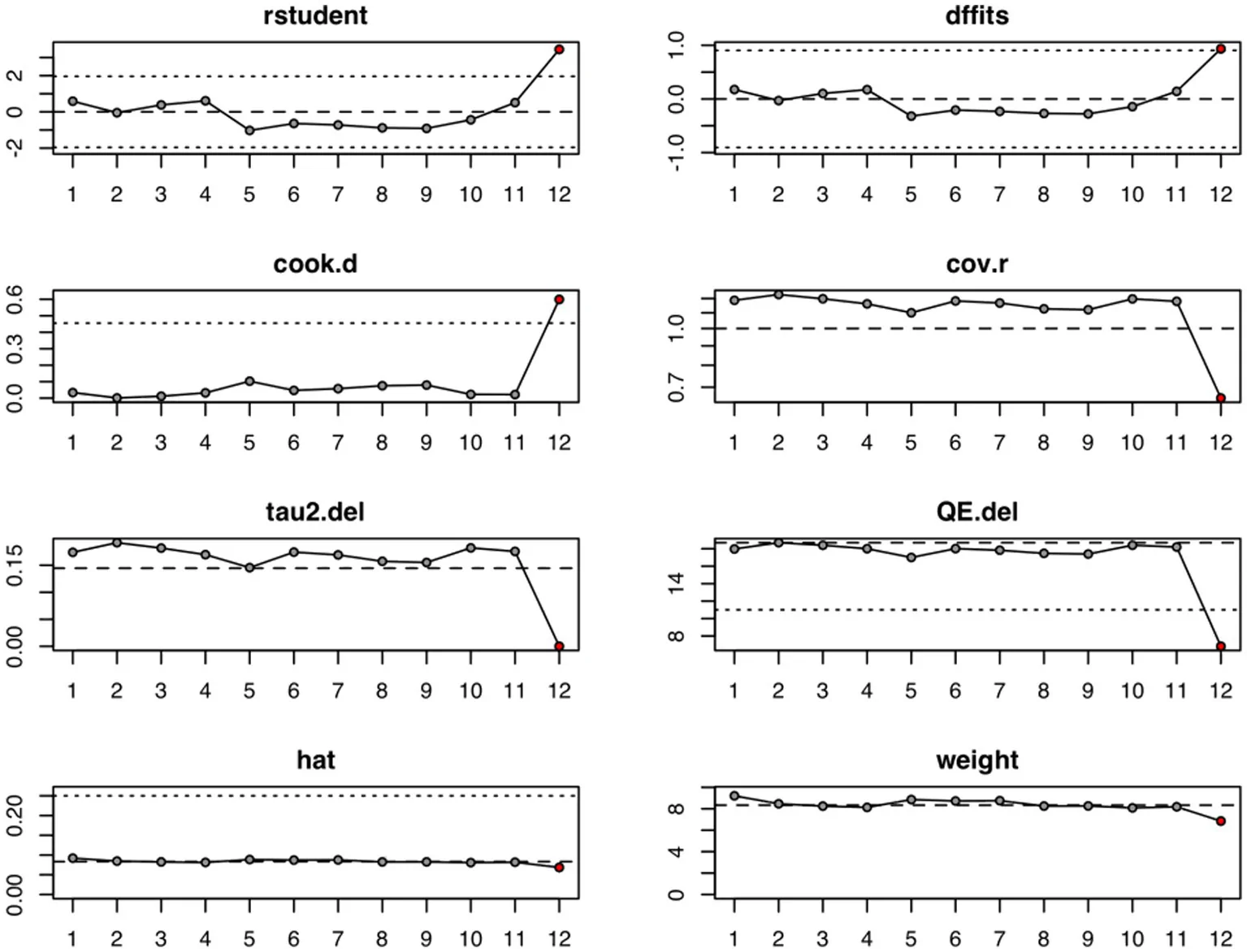
Funnel plot for life status outcomes.

Caution is warranted when interpreting these publication-bias diagnostics: the number of observed effect-size estimates for life status outcomes was limited. Under small-sample conditions, Egger’s regression, visual inspection of funnel plots, and the trim-and-fill method have limited statistical power. The absence of significant asymmetry does not allow complete exclusion of potential publication bias. Publication-bias diagnostics were not repeated for the sensitivity-analysis dataset after removal of high-influence effect-size points.

#### 3.3.5 Main effect of MGI on psychological outcomes in stroke patients

##### 3.3.5.1 Overall effect analysis

Before removing outliers, the three-level random-effects model showed an effect size of *g* = 1.7287 (95% CI: 1.0449 to 2.4125, *p* = 0.0002) for MGI on psychological outcomes in stroke patients, representing a large effect. Verification with the two-level model yielded g = 1.5989 (95% CI: 1.2084 to 1.9894, *p* < 0.001), indicating that MGI significantly improves psychological status (e.g., depressive mood, anxiety levels) (Figure 19).

**Figure 19:**
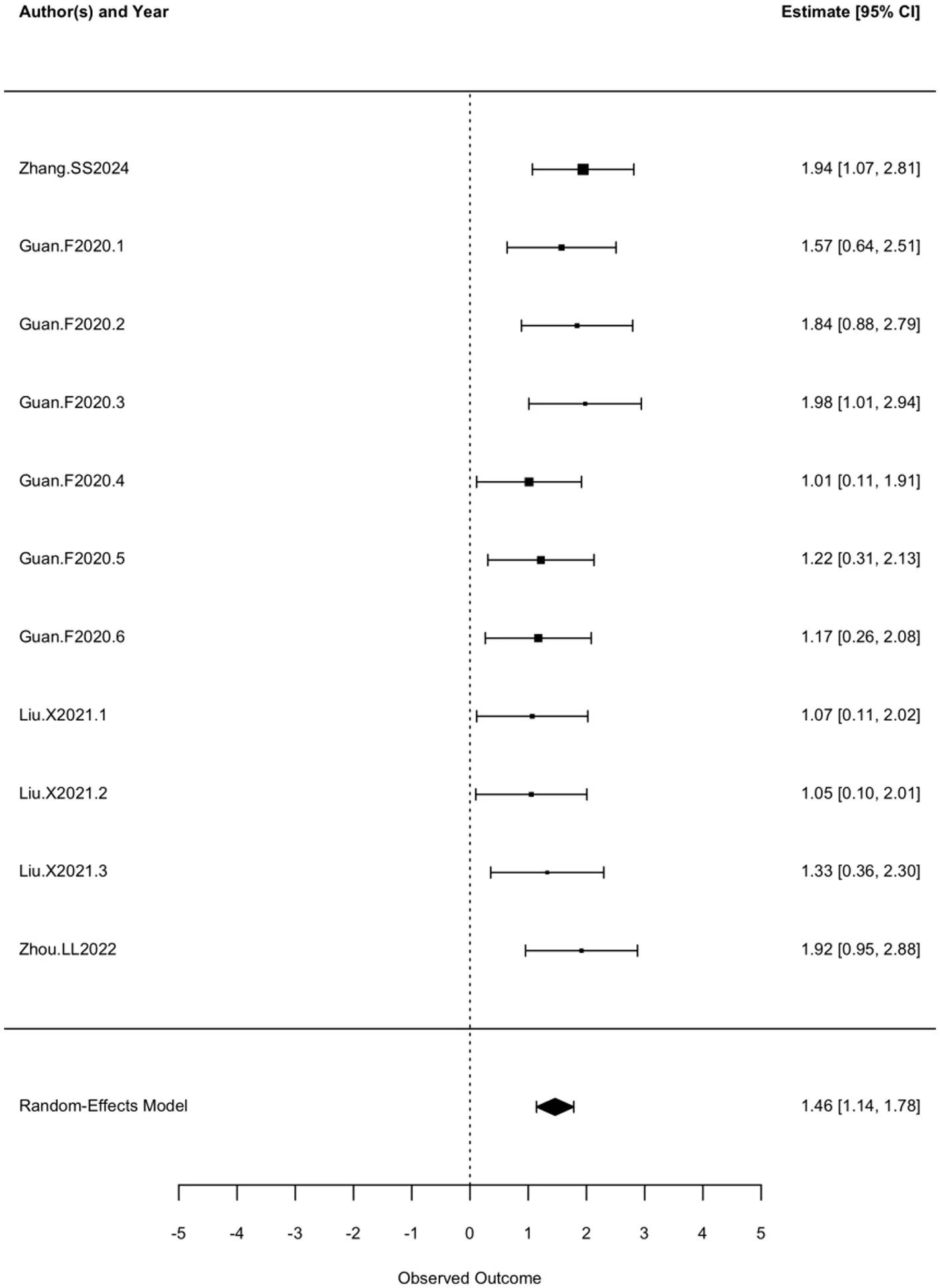
Forest plot of the primary main analysis for psychological outcomes.

##### 3.3.5.2 Influence analysis

The leave-one-out method identified Effect Size ID = 12 as an outlier (removal led to tau^2^ decreasing from 0.1445 to 0.0000 and I^2^ from 38.33 to 0.00%). After sequentially removing other effect sizes, the effect size fluctuated between 1.4055 and 1.5101, all *p*-values remained < 0.001, and heterogeneity did not increase significantly, indicating a stable main effect (Figure 20).

**Figure 20:**
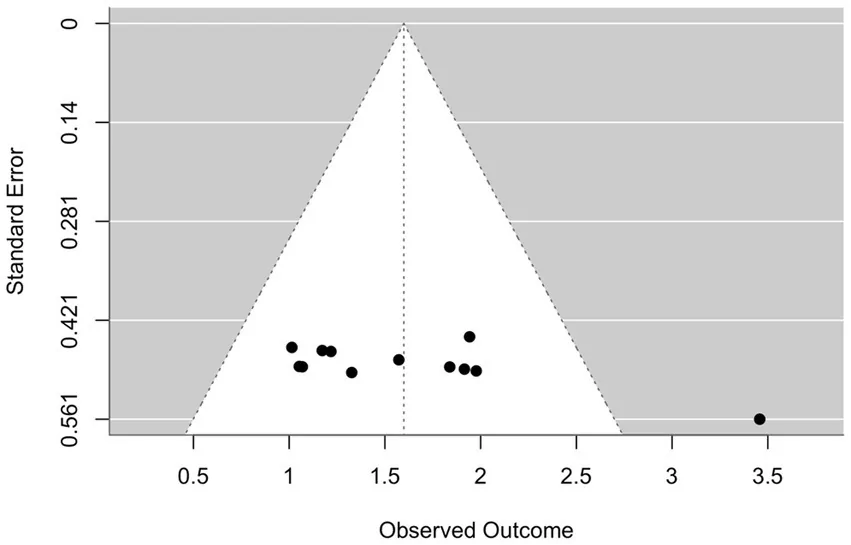
Influence analysis plot for psychological outcomes.

After removing outlier (Effect Size ID = 12), the three-level model effect size from this sensitivity analysis was *g* = 1.4616 (95% CI: 1.1417 to 1.7816, *p* < 0.0001), still a large effect. The two-level model result was consistent, suggesting stable intervention effects (Figure 21).

**Figure 21:**
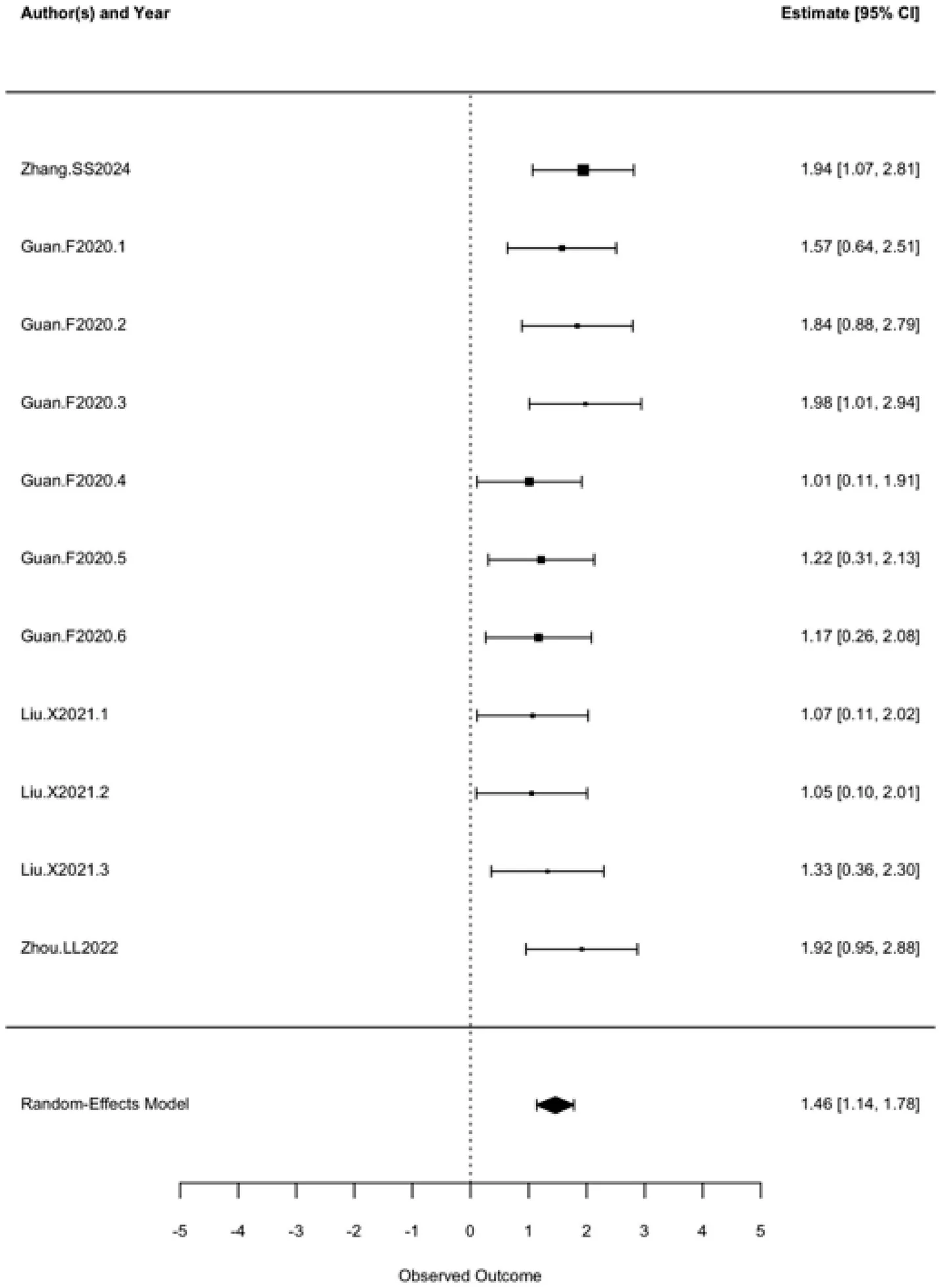
Forest plot of the sensitivity analysis for psychological outcomes.

##### 3.3.5.3 Heterogeneity analysis

Before outlier removal, heterogeneity testing showed *Q* = 18.6949 (df = 11, *p* = 0.0668), *I*^2^ = 38.33%, indicating moderate heterogeneity. Variance component decomposition: Level 2 variance σ^2^.1 = 0.0000 (*p* = 1.0000), Level 3 variance σ^2^.2 = 0.2824 (*p* = 0.1055), suggesting between-study variance was the primary source. Sampling variance accounted for 61.67%, between-study variance for 38.33% of the total variance. After outlier removal, heterogeneity disappeared: *Q* = 6.8193 (df = 10, *p* = 0.7424), *I*^2^ = 0.00%; both Level 2 and Level 3 variances were 0.0000, and sampling variance accounted for 100.00%.

##### 3.3.5.4 Publication bias assessment

Publication-bias assessment was performed on the primary main-analysis dataset retaining all effect-size estimates, including high-influence observations. Egger’s regression test for funnel-plot asymmetry yielded *t* = 2.7869 (df = 10, *p* = 0.0192); the intercept estimate as the standard error approached zero was *b* = −5.9054 (95% CI: −11.9040 to 0.0932). Statistically significant funnel-plot asymmetry was detected (Figure 22).

**Figure 22:**
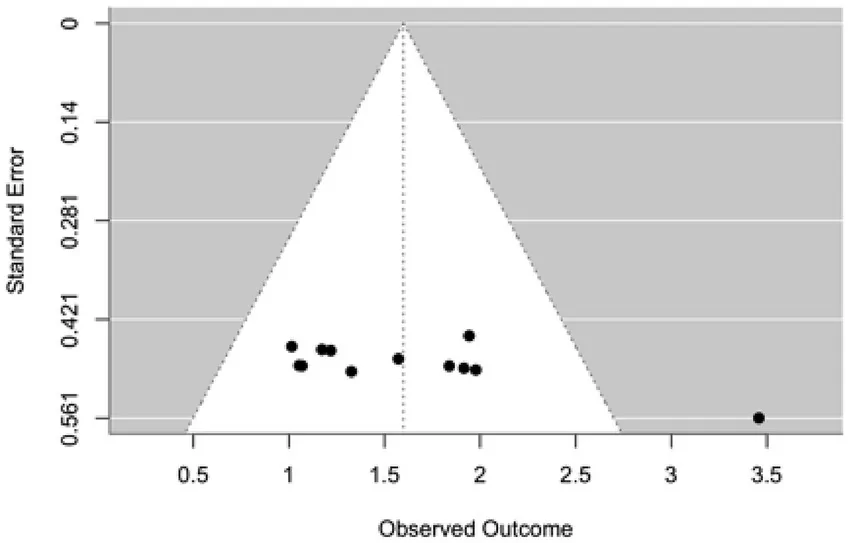
Funnel Plot for Psychological Outcomes.

Caution is warranted when interpreting these publication-bias diagnostics. Although the number of observed effect-size estimates for this outcome was relatively larger compared with other secondary outcomes, interpretation still requires prudence. Funnel-plot asymmetry can be caused by multiple factors apart from publication bias, including between-study heterogeneity, differences in participant characteristics, and variation in measurement instruments. A detected significant asymmetry signal cannot be solely attributed to publication bias. Publication-bias diagnostics were not repeated for the sensitivity-analysis dataset after removal of high-influence effect-size points.

### 3.4 Certainty of evidence (GRADE)

The GRADE assessment (34) indicated that the certainty of evidence was low for pain outcomes, very low for functional, sleep, life status, and psychological outcomes. The overall downgrading was attributable to multiple methodological limitations: ① Risk of bias: All included studies were assessed using RoB 2 at the outcome level. No individual study was rated as high risk of bias, but all studies had some concerns, with widespread deficiencies in allocation concealment and assessor blinding and no trials adopting intention-to-treat analysis. Since all outcomes were subjective or assessor-reported and susceptible to bias, these cumulative methodological limitations were judged as serious concerns and triggered one-level downgrade. ② Inconsistency, whereby residual heterogeneity existed before post-hoc outlier removal and observed variability could not be fully explained by predefined moderators. ③ Imprecision, with insufficient sample sizes failing to meet optimal information criteria for several outcomes. ④ Publication bias, owing to unperformed grey literature searches, potential language bias, and limited statistical power of asymmetry tests in small sample sets (see Table 5).

**Table 5:** GRADE certainty of evidence assessment.

Outcome	Included studies (n)	Certainty assessment					Certainty of evidence
		Risk of bias	Inconsistency	Indirectness	Imprecision	Publication bias	
Pain	11	serious^1^	Not serious	Not serious	Not serious	Serious^2^	Low
Function	7	serious^1^	Serious^3^	Not serious	Not serious	Serious^2^	Very low
Sleep	3	serious^1^	Serious^4^	Not serious	Serious^5^	Serious^2^	Very low
Life status	6	serious^1^	Serious^4^	Not serious	Serious^5^	Serious^2^	Very low
Psychological	4	serious^1^	Serious^4^	Not serious	Serious^5^	Serious^2^	Very low

GRADE judgements followed Cochrane GRADE handbook guidance for RCT evidence, starting from high baseline certainty. Judgments avoid simple arithmetic summation of downgrade levels. Not serious indicates limitations exist but do not meet the threshold for formal downgrading (only noted as limitations). Serious indicates a serious concern that triggered a one-level downgrade. No domain across all outcomes reached the threshold for very serious concern (two-level downgrade). For sleep, life status and psychological outcomes, serious concerns existed across three independent domains (inconsistency, imprecision, and publication bias); cumulative downgrading across these domains resulted in a final certainty rating of very low. ^1^ Risk of bias: Included studies were assessed using RoB 2 at the outcome level. No individual study was rated high risk of bias; all studies had some concerns, with widespread deficiencies in allocation concealment, assessor blinding, and no studies applied intention-to-treat analysis. Since all outcomes are subjective or assessor-reported and susceptible to bias, these cumulative methodological limitations were judged as serious concerns, triggering a one-level downgrade. ^2^ Publication bias: No gray literature search was performed and potential language bias risk existed. For outcomes with a small to moderate number of independent RCTs (e.g., *n* < 20), exploratory tests including funnel plot inspection, Egger’s test and trim-and-fill have low to moderate statistical power. Non-significant results of these exploratory tests cannot be interpreted as evidence of absent publication bias. ^3^ Inconsistency for motor-related functional outcomes: Moderate heterogeneity was observed before outlier removal, and residual variation in point estimates could not be fully explained. ^4^ Inconsistency for sleep quality, life status and psychological-mood outcomes: Heterogeneity existed in original analyses and was resolved only by post-hoc outlier removal. No subgroup or moderator documented within the retrospectively registered PROSPERO record could account for the original variability. Small study numbers further restricted exploration of heterogeneity sources. ^5^ Imprecision for sleep quality, life status and psychological-mood outcomes: Total sample size failed to meet optimal information size criteria, although confidence intervals excluded the null effect.

## 4 Discussion

This three-level meta-analysis systematically evaluated the multi-dimensional effects of MGI training on pain, function, sleep, life status, and psychological mood in stroke patients. It not only validated the clinical value of this therapy but also addressed core gaps in existing research through methodological innovation and expanded perspectives. The key findings and their innovative implications are further elucidated below in conjunction with previous research.

### 4.1 Efficacy and underlying mechanisms of motor-guided imagery for pain relief in stroke patients

Previous meta-analyses on the analgesic effect of motor imagery have yielded inconsistent conclusions. For instance, Braun et al. (9) included 7 randomized controlled trials (RCTs) and reported a significant moderate analgesic effect (SMD = −0.62). However, Thieme et al. (11) noted high heterogeneity among included studies (*I*^2^ = 68%) due to variations in pain type and intervention protocols, suggesting that the therapeutic effect remains to be confirmed. The present study overcomes these limitations through two methodological innovations. First, a three-level meta-analytic model was applied to account for the nested structure of multiple effect sizes within a single study, which allowed for precise partitioning of sampling variance, within-study variance, and between-study variance. This approach yielded a more accurate pooled effect size (*g* = −0.5240, 95% CI: −0.7788 to −0.2691), representing a moderate and robust effect. The three-level model revealed total heterogeneity of *Q* = 3.8803 (df = 14, *p* = 0.9961) with I^2^ = 0.00%, and variance decomposition indicated that 100% of the variability was attributable to sampling error, with negligible within-study and between-study variance. This resolved the issue of misestimated heterogeneity inherent in traditional two-level models that ignore non-independence of effect sizes. Leave-one-out influence analysis further demonstrated that the effect size ranged narrowly from −0.4942 to −0.5483 and remained statistically significant in all iterations, with no outlier driving the overall result, thus verifying the stability of the main effect.

Second, this study further performed stratified analyses according to pain subtypes. Both shoulder pain and mixed-type pain showed moderate improvement effects (shoulder pain: *g* = −0.569; mixed-type pain: *g* = −0.504), and the moderating effect did not reach statistical significance (*p* = 0.803). This finding suggests that motor-guided imagery training may be applicable to different types of post-stroke pain. Nevertheless, given the absence of statistically significant differences between subgroups, these findings should be interpreted as exploratory rather than confirmatory.

Further subgroup comparisons revealed that the analgesic effect was large among patients in the acute phase (disease duration ≤ 4 weeks; g = −0.912), whereas no significant effect was observed in the subacute phase (> 4 weeks; g = −0.268, *p* = 0.380). This trend is consistent with the theoretical hypothesis of stronger neuroplasticity in the early post-stroke period, whereby early-stage rehabilitation may yield greater benefits (1). Meanwhile, no statistically significant difference was detected between motor-guided imagery monotherapy and combined interventions (*p* = 0.710). This suggests that motor-guided imagery may have certain value for independent application, especially in clinical scenarios with limited resources or where complex combined regimens are inappropriate for patients.

The improvement in functional outcomes associated with motor-guided imagery may be attributed to the activation of motor imagery networks. Through repeated mental simulation of limb movements, motor imagery may enhance the efficiency of connections between the motor cortex and spinal motor neurons, thereby promoting motor function recovery (40, 41). Improvements in sleep may be related to the attenuation of the “pain–insomnia” vicious cycle following pain relief, as well as to the regulation of autonomic nervous function (42, 43). However, the included RCTs did not directly measure neuroimaging or autonomic indicators; therefore, the above mechanistic interpretations remain plausible speculations based on previous literature rather than empirical findings of the present meta-analysis.

For life status outcomes, the pooled effect size was *g* = 1.4944 (95% CI: 0.9467 to 2.0422, *p* = 0.0005), suggesting that motor-guided imagery training may improve patients’ activities of daily living and quality of life. Pain and functional impairment are important modifiable factors affecting the quality of life of stroke patients (44). Therefore, pain relief and functional improvement may jointly promote patients’ activity capacity and social participation, which is consistent with the hierarchical logic of the ICF framework, “Body Function → Activity → Participation” (16, 45).

Analysis of psychological indicators showed an effect size of g = 1.4616 (95% CI: 1.1417 to 1.7816, *p* < 0.0001), confirming that MGI effectively alleviates negative emotions such as anxiety and depression in stroke patients. Substantial evidence indicates that post-stroke somatic dysfunction and chronic pain are key factors in inducing and sustaining depressive mood, often intertwining to form a vicious cycle that is difficult to break (15). Therefore, by directly improving patients’ pain perception and motor function, MGI may fundamentally weaken the driving factors of this cycle, thereby indirectly improving psychological state. Neuroscientific research provides a deeper explanation: the neural circuits in the brain responsible for motor planning, execution, and reward motivation (e.g., the prefrontal-basal ganglia pathway) exhibit high anatomical overlap and functional integration with the neural networks regulating emotion (46). Targeted activation of the motor network (e.g., through motor imagery) may positively modulate emotional states by regulating the functional state of this shared circuitry.

Large improvement effects were observed for psychological mood outcomes (*g* = 1.4616, 95% CI: 1.1417 to 1.7816, *p* < 0.0001). Post-stroke pain, functional impairment and negative mood frequently interact and form a vicious cycle (15). Motor-guided imagery training may indirectly alleviate anxiety, depression and other emotional-disturbance problems by reducing pain perception, improving motor function, and strengthening patients’ sense of active engagement. Neuroscientific evidence also suggests functional integration among networks involved in motor planning, reward-related motivation, and emotion regulation (46, 47). Nevertheless, the present meta-analysis cannot provide direct empirical confirmation of these underlying neural pathways.

It should be noted that composite outcomes including function, life status, and psychological mood outcomes integrate multiple measurement indicators with distinct clinical connotations. Although sensitivity analyses supported the robustness of the overall pooled estimates, the clinical constructs represented by different scales and indicators are not entirely consistent. Caution is therefore required when interpreting the magnitude of these effect sizes.

### 4.2 Exploratory observations of intervention-related parameters

In terms of subgroup analyses, previous studies have shown substantial heterogeneity in motor-guided imagery intervention protocols, including training frequency, single-session duration, total exposure time, and forms of combined interventions (12, 13). Moderator analyses were performed using a three-level mixed-effects model. Although no statistically significant moderating effects were identified for variables such as age, sex, single-session duration, and total exposure time, several exploratory numerical trends were observed.

The subgroup with > 5 training sessions per week yielded a large analgesic effect size (*g* = −0.585, *p* = 0.004), whereas no statistically significant effect was detected in the subgroup with ≤5 sessions per week (*g* = −0.380, *p* = 0.097). Significant analgesic effects could be achieved with single-session training ≤30 min (*g* = −0.512, *p* = 0.013); longer single-session duration (> 30 min) demonstrated no additional benefit (*g* = −0.505, *p* = 0.020). A numerical tendency toward larger effect sizes was seen for the “high-frequency, short-duration” regimen, though overall moderator testing remained non-significant, and this remains a direction for future validation work rather than an established clinical recommendation (47).

Regarding disease duration and treatment course, patients in the acute phase (≤4 weeks) had a larger effect estimate (*g* = −0.912, *p* = 0.013), while the effect did not reach statistical significance for patients in the subacute phase (>4 weeks, *g* = −0.268, *p* = 0.380). Numerically larger effect sizes were also observed for the subgroup with an intervention course ≤ 4 weeks (*g* = −0.604, *p* = 0.008). These numerical observations point to potential signals for early-initiated and short-course interventions. Importantly, because the overall moderator test was not statistically significant, all subgroup-level observations are purely exploratory and hypothesis-generating, and cannot support definitive inferences regarding intervention superiority (48). Future large-sample RCTs are required to verify these preliminary signals.

For total exposure time, low-to-moderate total exposure (≤360 min) produced significant analgesic effects (*g* = −0.541, *p* = 0.042), and higher total exposure (>360 min) did not yield further therapeutic improvement (*g* = −0.495, *p* = 0.007). This merely represents a numerical subgroup signal; no definitive inference can be drawn that motor-guided imagery training relies more on timely initiation and sufficient training frequency instead of prolonged single-session or total training volume. Given the small number of studies within each subgroup, future large-sample RCTs are required to verify this preliminary observation.

Subgroup analysis stratified by measurement tool was performed for functional outcomes to verify the rationality of pooling different clinical indicators into a composite functional outcome. The results demonstrated that subgroup effect sizes for FMA, shoulder range of motion, swelling volume, and the GQOLI-74 physical subscale were all positive and statistically significant, with no significant difference in effect sizes across subgroups (*p* = 0.251). These findings indicate that although these indicators assess distinct dimensions, they consistently reflect the improvement in post-stroke upper-limb function induced by MGI, and the statistical results support the pooling strategy adopted for the composite functional outcome. Meanwhile, residual heterogeneity remained in the model, suggesting unexplained clinical variation among functional outcomes. We downgraded the evidence for inconsistency in the GRADE assessment and interpreted the findings with caution.

### 4.3 Limitations and future perspectives

This study has several limitations. First, the number of studies included for some outcome indicators (e.g., sleep, psychology) was relatively small, which may affect the stability of moderator analyses. For pain and function outcomes, no statistically significant moderators were found (all *p* > 0.05), precluding multiple moderator regression. For sleep, life status, and psychological outcomes, the limited number of studies prevented moderator analysis altogether. Second, all included studies exhibited overall some concerns in methodological quality according to the RoB 2 tool. Most studies lacked adequate allocation concealment, no trials applied intention-to-treat analysis, only two of the included studies implemented assessor blinding, introducing a risk of measurement bias. The lack of grey literature search may represent a potential limitation, which could introduce a certain risk of publication bias. Third, part of the effect-size dataset was derived from multiple secondary publications of a single parent trial. Although the three-level meta-analysis was applied to handle the non-independence of these effect sizes, these observations do not constitute fully independent samples, which imposes certain limitations on the estimation of between-study heterogeneity; therefore, conclusions should be interpreted with caution. Fourth, Identification of influential effect-size observations was an exploratory post-hoc statistical diagnostic procedure. Merely detecting influential points via statistical metrics does not provide sufficient grounds to exclude eligible study data from primary analysis. Analyses after removing influential observations should therefore only be interpreted as sensitivity-based robustness references, rather than primary evidence for drawing conclusions. Finally, this systematic review exclusively included randomized controlled trials. Observational studies, case reports and other non-RCT study designs were not eligible for inclusion. Although RCTs yield high-quality evidence for intervention effectiveness, real-world insights derived from observational or case-based research could not be captured under the current eligibility criteria. This constitutes an inherent limitation of the present evidence base.

Future research should advance in three directions. First, methodologically, multicenter, large-sample RCTs, particularly focusing on acute stroke patients, should be conducted to verify the precise efficacy of optimized dosing regimens like “high-frequency, short-duration.” This addresses the urgent call within the international stroke rehabilitation research consensus to determine optimal treatment parameters during key recovery periods (49). Second, regarding mechanism exploration, advanced neuroimaging techniques such as functional magnetic resonance imaging (fMRI) should be integrated to move beyond behavioral assessments. Research should directly investigate how MGI modulates functional connectivity and activity patterns within core brain networks involved in pain processing, particularly key nodes of the “pain matrix” like the insula and anterior cingulate cortex. Elucidating how this therapy affects these brain regions, which are closely associated with the affective and cognitive dimensions of pain (50), will be crucial for uncovering its central analgesic mechanisms. Finally, concerning effect evaluation, well-designed long-term follow-up studies (e.g., 6 months, 1 year) are essential to assess whether the intervention effects are sustained and ultimately translate into long-term functional independence and quality-of-life improvements for patients. This is a critical step for translating stroke rehabilitation research into clinical practice (51).

In summary, through methodological innovation using a three-level model, stratified analysis by pain subtype, and construction of a multidimensional outcome evaluation system, this study not only precisely quantified the analgesic effect of MGI but also confirmed its value in multi-system synergistic improvement. It also clarified optimized intervention parameters. By overcoming limitations in methodology, evaluation dimensions, and parameter quantification present in previous research, this study provides higher-level evidence to support the standardized application of this therapy in stroke rehabilitation.

## 5 Conclusion

Motor Guided Imagery training may tentatively alleviate pain and improve motor function, sleep quality, life status, and psychological mood among stroke patients. Exploratory moderator analyses revealed numerical trends toward larger effect sizes for interventions initiated in the acute phase and for high-frequency, short-duration regimens. Since overall moderator tests did not reach statistical significance, these findings are hypothesis-generating rather than definitive clinical conclusions and require confirmation from future well-designed randomized controlled trials.

Motor Guided Imagery training is easy to administer and not constrained by patients’ limb motor capacity and can serve as an important adjunctive rehabilitation approach for stroke patients. It may help alleviate pain and improve functional performance, sleep, life status, and psychological mood outcomes among stroke survivors. Current evidence supports its potential role as an adjunctive intervention for stroke rehabilitation, yet cautious interpretation is required regarding optimal intervention frequency, single-session duration, total treatment course, and target patient populations. Future well-designed randomized controlled trials with larger sample sizes, standardized intervention reporting, and adequate follow-up periods are warranted. Neuroimaging or mediation analyses are also needed to further validate its underlying mechanisms of action.

## Data Availability

The original contributions presented in the study are included in the article/Supplementary material, further inquiries can be directed to the corresponding author.
