# Supplementary material for: Impact of motor guided imagery therapy on stroke patients with pain symptoms: a three-level meta-analysis and systematic review of randomized controlled trials
Source: Front Neurol. 2026 Oct 6;17:1920523. doi: 10.3389/fneur.2026.1920523 (PMC13682273; doi:10.3389/fneur.2026.1920523)
Supplement: Supplementary file 1 [file Supplementary_file_1.docx]

**Table 1. Systematic Literature Search Strategy**

| Database | Search Steps |
| --- | --- |
| PubMed and The Cochrane Library | #1 Stroke[Title/Abstract] OR "Cerebrovascular accident"[Title/Abstract] OR "Hemorrhagic stroke"[Title/Abstract] OR "Intracerebral hemorrhage"[Title/Abstract] OR "Subarachnoid hemorrhage"[Title/Abstract] OR "Cerebrovascular disorder"[Title/Abstract] OR "Ischemic stroke"[Title/Abstract] OR Post-stroke[Title/Abstract] OR "Cerebral infarction"[Title/Abstract] OR CVA[Title/Abstract] OR ICH[Title/Abstract] OR "Cerebral Stroke"[Title/Abstract] OR "Cerebrovascular Apoplexy"[Title/Abstract] OR "Brain Vascular Accident"[Title/Abstract] OR "Cerebrovascular Stroke"[Title/Abstract] OR Apoplexy[Title/Abstract] OR "Acute Stroke"[Title/Abstract] OR "Acute Cerebrovascular Accident"[Title/Abstract] OR Hemiplegia[Title/Abstract] OR Hemiparesis[Title/Abstract] OR Hemiparetic[Title/Abstract] OR "Stroke"[Mesh]  #2 "Motor imagery"[Title/Abstract] OR "Guided motor imagery"[Title/Abstract] OR "Kinesthetic imagery"[Title/Abstract] OR "Visual motor imagery"[Title/Abstract] OR "Mental practice"[Title/Abstract] OR "Imagery-based intervention"[Title/Abstract] OR "Motor imagery training"[Title/Abstract] OR "Motor Imagery Therapy"[Title/Abstract] OR "Mental imagery"[Title/Abstract] OR "Visual imagery"[Title/Abstract] OR "Mental training"[Title/Abstract] OR "Mental rehearsal"[Title/Abstract] OR "Motor Visualization"[Title/Abstract] OR "Exercise-Guided Imagery"[Title/Abstract] OR "Kinetic-Imaginal Engagement"[Title/Abstract] OR "Imagery, Psychotherapy"[Mesh]  #3 Pain[Title/Abstract] OR "Chronic pain"[Title/Abstract] OR "Musculoskeletal pain"[Title/Abstract] OR "Neuropathic pain"[Title/Abstract] OR "Postoperative pain"[Title/Abstract] OR "Central Post-Stroke Pain"[Title/Abstract] OR CPSP[Title/Abstract] OR "Shoulder Pain"[Title/Abstract] OR "Spastic Pain"[Title/Abstract] OR "Pain intensity"[Title/Abstract] OR "Pain frequency"[Title/Abstract] OR "Pain"[Mesh]  #4 Randomized controlled trial[Title/Abstract] OR Controlled clinical trial[Title/Abstract] OR Random allocation[Title/Abstract] OR placebo[Title/Abstract]  #5 #1 AND #2 AND #3 AND #4 |
| Web of Science | #1 TS=(Stroke OR "Cerebrovascular accident" OR "Hemorrhagic stroke" OR "Intracerebral hemorrhage" OR "Subarachnoid hemorrhage" OR "Cerebrovascular disorder" OR "Ischemic stroke" OR Post-stroke OR "Cerebral infarction" OR CVA OR ICH OR "Cerebral Stroke" OR "Cerebrovascular Apoplexy" OR "Brain Vascular Accident" OR "Cerebrovascular Stroke" OR Apoplexy OR "Acute Stroke" OR "Acute Cerebrovascular Accident" OR Hemiplegia OR Hemiparesis OR Hemiparetic)  #2 TS=("Motor imagery" OR "Guided motor imagery" OR "Kinesthetic imagery" OR "Visual motor imagery" OR "Mental practice" OR "Imagery-based intervention" OR "Motor imagery training" OR "Motor Imagery Therapy" OR "Mental imagery" OR "Visual imagery" OR "Mental training" OR "Mental rehearsal" OR "Motor Visualization" OR "Exercise-Guided Imagery" OR "Kinetic-Imaginal Engagement")  #3 TS=(Pain OR "Chronic pain" OR "Musculoskeletal pain" OR "Neuropathic pain" OR "Postoperative pain" OR "Central Post-Stroke Pain" OR CPSP OR "Shoulder Pain" OR "Spastic Pain" OR "Pain intensity" OR "Pain frequency")  #4 #1 AND #2 AND #3 NOT Preprint Citation Index |
| Embase | #1 "stroke"/exp OR stroke OR "cerebrovascular accident"/exp OR "cerebrovascular accident" OR "hemorrhagic stroke"/exp OR "hemorrhagic stroke" OR "intracerebral hemorrhage"/exp OR "intracerebral hemorrhage" OR "subarachnoid hemorrhage"/exp OR "subarachnoid hemorrhage" OR "cerebrovascular disorder"/exp OR "cerebrovascular disorder" OR "ischemic stroke"/exp OR "ischemic stroke" OR "post stroke"/exp OR "post stroke" OR "cerebral infarction"/exp OR "cerebral infarction" OR "cva"/exp OR cva OR ich OR "cerebral stroke" OR "cerebrovascular apoplexy" OR "brain vascular accident"/exp OR "brain vascular accident" OR "cerebrovascular stroke" OR "apoplexy"/exp OR apoplexy OR "acute stroke" OR "acute cerebrovascular accident" OR "hemiplegia"/exp OR hemiplegia OR "hemiparesis"/exp OR hemiparesis OR hemiparetic:ab,ti  #2 "guided imagery"/exp OR "guided imagery" OR "guided motor imagery" OR "kinesthetic imagery" OR "visual motor imagery" OR "mental practice" OR "motor imagery training" OR "motor imagery therapy" OR "mental imagery" OR "visual imagery" OR "mental training" OR "mental rehearsal" OR "motor visualization" OR "exercise-guided imagery" OR "kinetic-imaginal engagement":ab,ti  #3 "pain"/exp OR pain OR "chronic pain"/exp OR "chronic pain" OR "musculoskeletal pain"/exp OR "musculoskeletal pain" OR "neuropathic pain"/exp OR "neuropathic pain" OR "postoperative pain"/exp OR "postoperative pain" OR "central post-stroke pain" OR CPSP OR "shoulder pain" OR "spastic pain" OR "pain intensity" OR "pain frequency":ab,ti  #4 #1 AND #2 AND #3 |
| CNKI | 主题 =（运动引导想象 + 运动想象 + 运动想象引导干预 + 动作想象 + 动觉想象 + 引导式运动想象 + 运动想象疗法 + 基于想象的干预 + 运动想象训练 + 心理练习 + 运动可视化）AND 主题 =（脑卒中 + 脑血管意外 + 缺血性脑卒中 + 出血性脑卒中 + 卒中后 + 脑中风 + 脑梗死 + 脑出血 + 脑卒中后偏瘫 + 中风 + 脑血管疾病）AND 主题 =（疼痛 + 慢性疼痛 + 肌肉骨骼痛 + 术后疼痛 + 神经病理性疼痛 + 疼痛强度 + 疼痛频率 + 中枢性疼痛 + 肩痛 + 痉挛性疼痛 + 头痛 + 内脏痛 + 外周痛 + 压疮相关疼痛） |
| Wan fang, CBM and VIP | 主题 =（运动引导想象 OR 运动想象 OR 运动想象引导干预 OR 动作想象 OR 动觉想象 OR 引导式运动想象 OR 运动想象疗法 OR 基于想象的干预 OR 运动想象训练 OR 心理练习 OR 运动可视化）AND 主题 =（脑卒中 OR 脑血管意外 OR 缺血性脑卒中 OR 出血性脑卒中 OR 卒中后 OR 脑中风 OR 脑梗死 OR 脑出血 OR 脑卒中后偏瘫 OR 中风）AND 主题 =（疼痛 OR 慢性疼痛 OR 肌肉骨骼痛 OR 术后疼痛 OR 神经病理性疼痛 OR 疼痛强度 OR 疼痛频率 OR 中枢性疼痛 OR 肩痛 OR 痉挛性疼痛 OR 头痛 OR 内脏痛 OR 外周痛 OR 压疮相关疼痛 |
